# Nonstructural Protein 1 of Influenza A (NS1A) Demonstrates Strain-Specific dsRNA Binding Capabilities

**DOI:** 10.1021/acsinfecdis.4c00882

**Published:** 2025-03-13

**Authors:** Veronica A. Smith, Aubrey R. Schall, John W. Tomsho

**Affiliations:** Saint Joseph’s University, Department of Chemistry & Biochemistry, University City Campus, 600 South 43rd Street, Philadelphia, Pennsylvania 19104, United States

**Keywords:** Influenza A, Spanish Flu, H1N1, H3N2, dsRNA

## Abstract

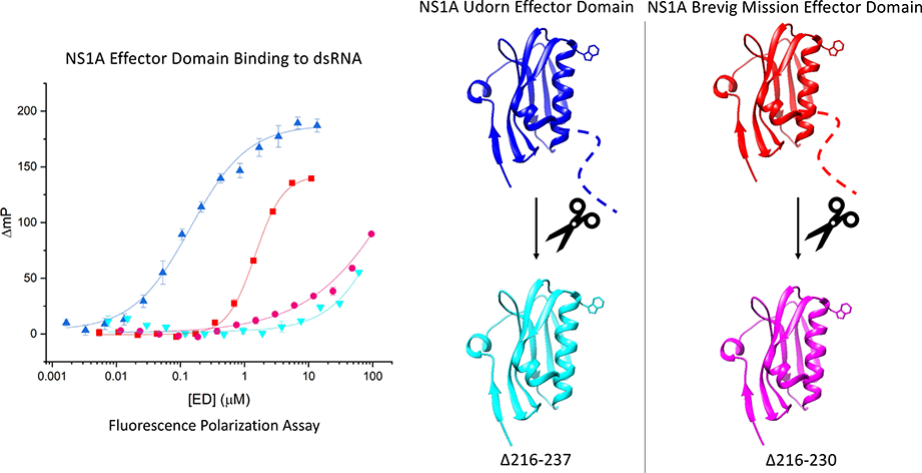

Nonstructural protein 1 of influenza A (NS1A) is a key virulence factor produced inside host cells infected with Influenza A Virus (IAV) and consists of an N-terminal dsRNA binding domain (RBD) and a C-terminal effector domain (ED), joined by a flexible linker. While NS1A is a highly promiscuous protein with a number of intracellular functions, its primary function is nonspecific dsRNA binding that enables influenza to evade our innate immune system. For this reason, NS1A has long been proposed as a potential drug target. Previous research in the field has demonstrated the necessity of dimer formation through the RBD to enable dsRNA binding, which is further enhanced by oligomerization through ED interactions. However, there has been minimal exploration of potential strain-specific effects on dsRNA binding. Most existing studies are limited to the A/Udorn/307/1972 strain, often with a C-terminal tail deletion. Here we utilize fluorescence polarization (FP) paired with fluorescence-based electrophoretic mobility shift assays (fEMSA) to characterize the dsRNA binding properties of NS1A from the H1N1 strain responsible for the 1918 “Spanish Flu” with an intact C-terminal tail. We show that A/Brevig Mission/1/1918 NS1A contains specific residues in the RBD that enhance dsRNA binding. We further demonstrate that both Brevig Mission and Udorn NS1A bind directly to dsRNA through the highly basic C-terminal tail of the ED. These novel binding interactions may have contributed to the increased pathogenicity of the 1918 flu pandemic and may have implications for NS1A-targeted antivirals.

Influenza is a serious public health threat, resulting in an estimated average of 41 million infections and 51,000 deaths between 2010 and 2023 in the US alone.^1^ Globally, ∼650,000 deaths are attributable to influenza infection each year.^2^ Since the turn of the 20th century, there have been four influenza pandemics (1918, 1957, 1968, and 2009). By far, the 1918 “Spanish Flu” pandemic was responsible for the highest estimated death toll, which is conservatively estimated at 50 million.^3^

The primary method of mitigation of the public health impact of influenza infections is prevention via annual vaccinations based on predicted seasonal flu strains.^4^ Postinfection, there are currently six FDA-approved treatments belonging to three different drug classes. The first class of anti-influenza treatments are M2 ion channel inhibitors, amantadine and rimantadine, however due to widespread resistance these treatments are no longer in use.^5−7^ The second class is composed of the neuraminidase inhibitors zanamivir, oseltamivir, and peramivir, which prevent the release of the newly formed viral particle.^5,8^ Despite the widespread use of these drugs, viral resistance can be easily achieved by the mutation of a single amino acid in the glycoprotein.^9^ Finally, in 2018 a first-in-class endonuclease inhibitor, baloxavir, was approved.^10^ However, while clinical trials indicated that this treatment resulted in a more rapid decline in viral load than oseltamivir, 10% of patients in phase 3 trials had strains with mutations which conferred reduced susceptibility to the drug.^11^ Based on the ability of influenza to rapidly gain resistance to the limited number of available therapeutics, the need for additional treatments and targets is clear.

One such target that has been of interest for a number of years is nonstructural protein 1 of influenza A (NS1A), a key virulence factor which is the nonstructural viral protein expressed in infected cells.^12^ NS1A is comprised of an N-terminal RNA binding domain (RBD) which is joined by a flexible linker region to a C-terminal effector domain (ED), the latter of which contains a disordered C-terminal tail of variable length, Figure 1.^13,14^ This protein dimerizes first through the RBD and can then form oligomers through ED interactions with neighboring dimer units, which is primarily facilitated by a key tryptophan residue, W187, on helix 3 of the ED.^15−19^ NS1A performs a variety of roles in the cell, including dsRNA sequestration through the RBD and extensive binding to various intracellular proteins, which occurs primarily through the ED.^20−22^

**Figure 1:**
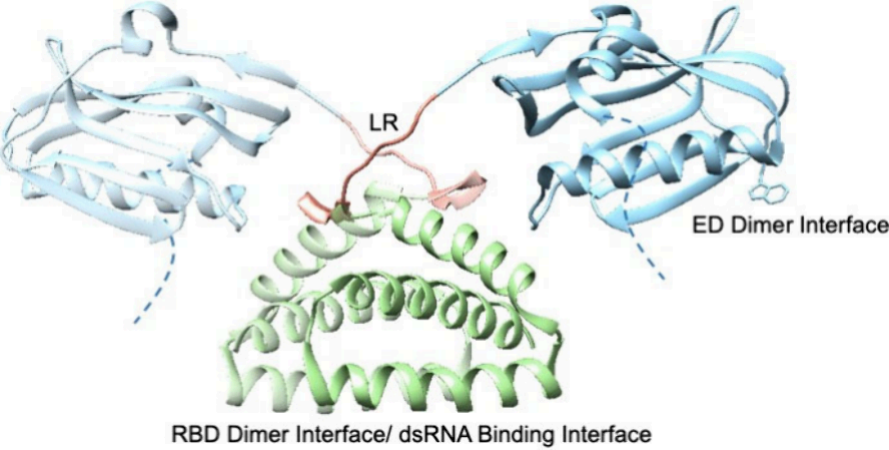
Dimeric NS1A. N-terminal RBD in green, LR in salmon, and ED in light blue with the key W187 residue shown. The unresolved disordered ED C-terminal tail shown as a blue dashed line. Second monomer unit set transparent for clarity and regions of interest are labeled. PDB# 4OPH.

Although NS1A overall remains a highly conserved protein, there are variations between influenza strains. While some research toward understanding strain specific function has been conducted regarding intracellular protein binding and variations in the linker region, the dsRNA binding properties of NS1A are largely excluded from these studies, and have not been fully evaluated.^16,17,23−27^ The vast majority of dsRNA binding studies have been conducted only on the A/Udorn/307/1972 (Udorn) strain, leaving the possibility that dsRNA binding activity can vary between strains. Additionally, the C-terminal tail region of the ED is frequently excluded due to its propensity to decrease protein solubility.^19^ These gaps in knowledge may have significant implications on the current understanding of NS1A function during viral infection.

When replication of a virus lacking NS1 was assessed, replication was inhibited up to three log fold.^28^ A similar three log fold reduction in viral replication was observed in a virus containing an R38A mutation in the RBD of NS1A.^20^ This mutant virus also demonstrated a three log fold increase in susceptibility to interferon-β (INF-β).^20^ Because this mutation also results in attenuated dsRNA binding, it was concluded that decreased dsRNA binding result in increased sensitivity to INF-β.^20^ Therefore, inhibition of NS1A’s dsRNA binding may aid in the ability of the innate immune system to more effectively clear viral infection. Because NS1A is not essential to the viral life cycle, it is less likely to develop resistance to an inhibitor and is therefore attractive as a new drug target. However, there are currently no therapeutics on the market for this target.

The main biochemical function of the RBD is to bind to dsRNA in a nonspecific manner, which blocks the 2′-5′- oligo (A) synthetase/RNase L pathway, consequently shielding the virus from our innate immune response.^20^ The binding of the RBD to dsRNA is reported to require both its dimeric structure, as well as a number of basic residues along the second α helix, with only R38 being absolutely essential for RNA binding activity.^29,30^ The RBD alone binds to short dsRNA with an affinity of ∼1 μM, although the reported values vary from 76 nM to 25 μM.^18,30−33^ As the length of the target dsRNA is increased, the affinity of the RBD for the dsRNA also increases.^29^

While the ED reportedly does not directly interact with dsRNA, it is necessary for enabling the full-length protein to bind to dsRNA in a cooperative manner.^18,34^ The tryptophan-mediated alpha helical ED dimer interface implicated in dsRNA sequestration, as these dimerization interactions allow for the formation of a tube-like structure around the dsRNA. When W187 was mutated to an arginine, the affinity of full-length NS1A for dsRNA was observed to be reduced 2-fold.^18^ Notably, this tryptophan residue is essential for not only ED dimerization, but also for interaction with the 30-kDa subunit of the cleavage and polyadenylation specificity factor (CPSF-30) which is one of the mechanisms by which NS1A inhibits host antiviral response.^18,19,35^ By virtue of sharing a binding interface facilitated by W187, ED dimerization and binding to CPSF-30 are mutually exclusive functions.^16^

While the importance of dsRNA binding by NS1A to support viral infection is clear, the current body of knowledge characterizing the nature of the interaction is limited by a lack of strain-specific information. Therefore, our objective was to characterize the interaction between the NS1A protein from the Brevig Mission H1N1 strain, responsible for the 1918 “Spanish Flu,” and dsRNA.

Herein, we describe the use of fluorescence polarization (FP) assays paired with fluorescence-based electrophoretic mobility shift assays (fEMSA) to quantify the binding of Brevig Mission NS1A to dsRNA. Our studies found that Brevig Mission NS1A binds to dsRNA directly through the ED, primarily mediated by the C-terminal tail, and additional residues in the RBD. Following the observation of this novel dsRNA binding, we sought to elucidate the residues or regions which contribute to these novel modes of binding, and subsequently created a library of mutants to test residues of interest. Through these studies, we demonstrate strain specific dsRNA binding between Brevig Mission and Udorn NS1A. These results potentially lend insight into the increased pathogenicity of the 1918 “Spanish Flu,” and may have implications for NS1A-targeted antivirals.

## Results and Discussion

### NS1A-RBD/dsRNA Binding Assays

**Figure 2:**
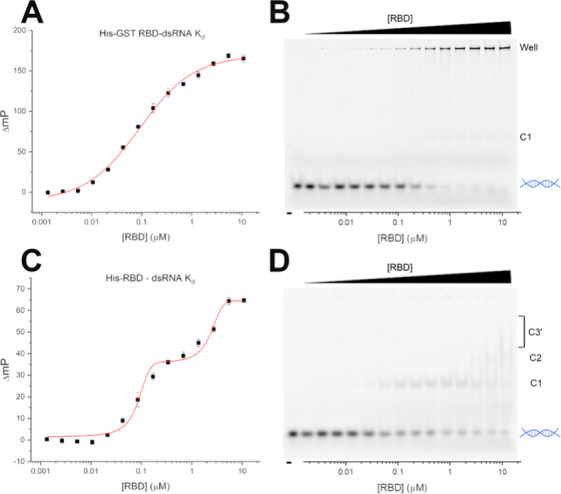
Binding affinity of Brevig Mission RBD for dsRNA. (A, B) Paired fluorescence polarization data for binding affinity of His-GST-RBD (*K*_d_ = 125 ± 9 nM) and fEMSA assay showing increased dsRNA binding with increasing His-GST-RBD concentration (*K*_d_ = 180 ± 42 nM). Data was plotted in OriginPro 2021b and fit to a logistic curve to obtain binding affinity. (C, D) Paired fluorescence polarization data for binding affinity of His-RBD (*K*_d_ = 83 ± 11 nM and 2160 ± 460 nM) and fEMSA assay showing increased dsRNA binding with increasing His-RBD concentration (*K*_d_ = 55 ± 16 nM). Data was plotted in OriginPro 2021b and fit to a biphasic (FP) or logistic (fEMSA) curve to obtain binding affinity. Leftmost lane is RNA only. Free dsRNA is depicted by blue helix on the right, with RBD dimer-dsRNA complexes shifted above, where C1 and C2 are distinct complexes while C3′ presents as a streak, indicating less stable complex formation. Data shown are representative of three independent experiments while values are presented as averages with standard deviations. fEMSA fitting data shown in Figure S28.

In order to evaluate the binding affinity of Brevig Mission NS1A RBD for dsRNA, the fluorescence polarization (FP) assay previously established by Cho et al.^33^ was paired with a fluorescence-based electrophoretic mobility shift assay (fEMSA) to serve as a secondary confirmation of our results. For these experiments, the same 16-bp dsRNA as Cho et al. was utilized, which was doubly labeled at the 5′ end with FITC (FAM-dsRNA). In our initial experiment, the binding of WT GST-tagged RBD (His-GST-RBD, residues 1–85) to FAM-dsRNA was determined to be 125 ± 9 nM (Figure 2A), which agrees well with the current body of literature.^18,30−33,36^ This value was well supported by that obtained by quantifying the free dsRNA from the fEMSA (180 ± 42 nM, Figure 2B).

Because GST is known to dimerize, assays were conducted to verify that this tag was not responsible for the obtained affinity or the binding stoichiometry, although Cho et al. have previously demonstrated that the GST tag does not directly bind to dsRNA.^33^ To do this, a His-RBD (residues 1–85) construct was created. Interestingly, FP of this construct resulted in a biphasic curve with binding affinities of 83 ± 11 nM and 2160 ± 460 nM (Figure 2C and 2D). This biphasic model was supported by the fEMSA, which showed evidence of at least two distinct RBD-dsRNA complexes. This observation may be explained by the occurrence of various NS1A:dsRNA stoichiometric complexes and this phenomenon will be addressed in detail below. The streaky nature of the bands above the second band at the highest concentrations may indicate the presence of an unstable NS1A:dsRNA complex.

Quantification of the free dsRNA in this assay resulted in an affinity of 55 ± 16 nM, corresponding well with the affinity obtained for the first transition. Because quantification is performed only on the free dsRNA, the second transition is not captured in this data. These results indicate that the GST tag does not significantly contribute to the observed RBD dimer-dsRNA binding, although it does appear to stabilize higher order complex formation. For the rest of our study, we elected to utilize the His-GST tagged constructs due to their larger size which is favorable for increased signal intensity in FP assays, and also because this allows for a more direct comparison with recent studies which utilized short dsRNA.^33,36^

### Neither Dimerization nor the R38/K41 Residues Are Essential for dsRNA Binding of Brevig Mission NS1A

**Figure 3:**
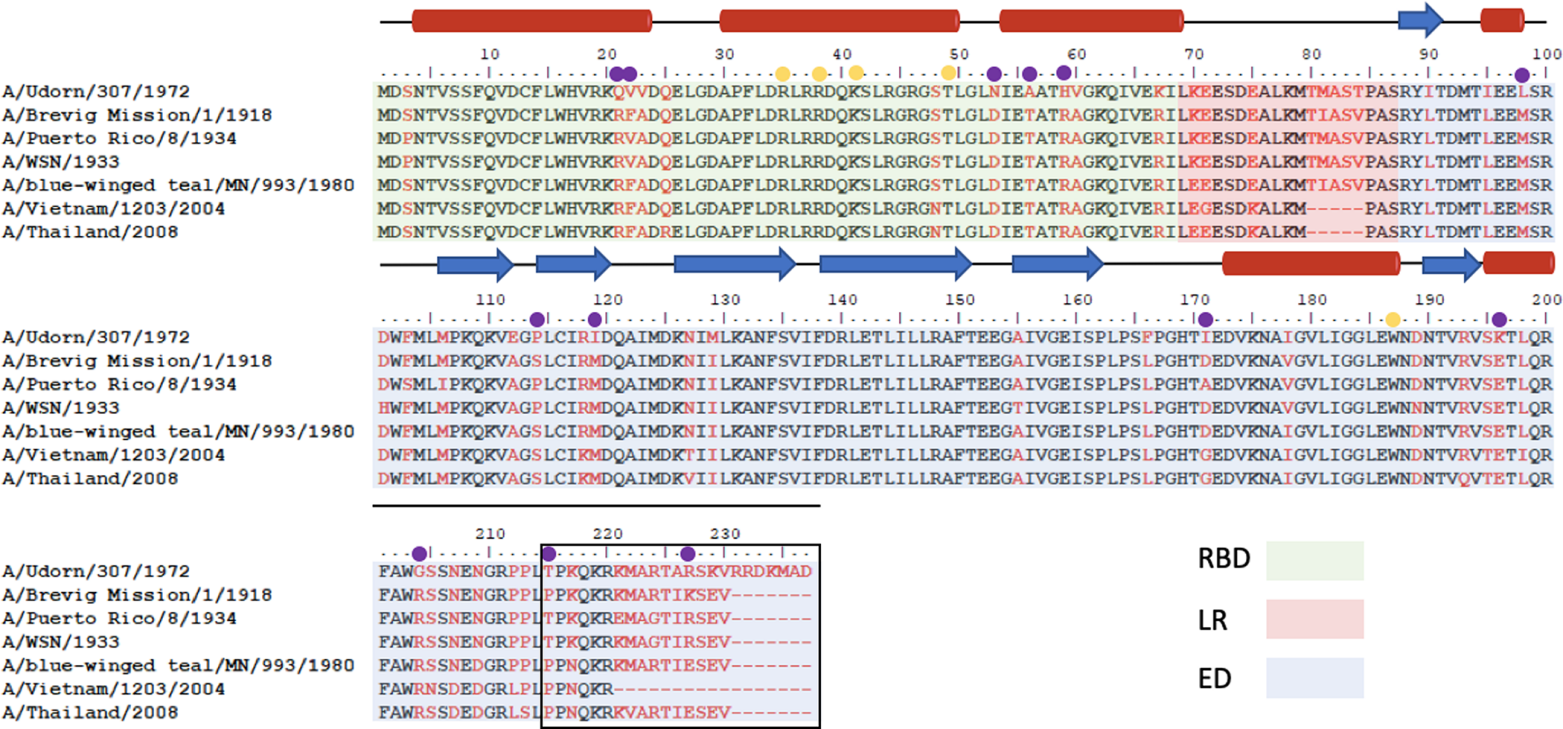
Multiple sequence alignment of commonly studied NS1A strains. Sequences were obtained from NCBI and aligned using BioEdit. RBD region highlighted in green, linker region highlighted in pink, ED highlighted in blue. Alpha helices indicated by red tubes; beta sheets indicated by blue arrows. Nonconserved residues highlighted in red. Residues selected for mutation for strain comparison are indicated by purple circles, while “control” mutants are indicated by yellow circles. The commonly deleted C-terminal tail residues are boxed in black.

The necessity of dimerization of the RBD for dsRNA binding has been established since 1999, and has since been reitterated.^29,30,32^ Because these studies were conducted on the Udorn strain, we sought to conduct analogous experiments for the Brevig Mission strain. In order to test whether dimerization of the RBD of Brevig Mission NS1A is necessary for dsRNA binding, both an RBD-only (1–85) and a full-length (1–230) construct containing a R35A mutation (His-GST-RBD R35A and His-GST-NS1A R35A, respectively) were created (Figure 3). Disruption of dimerization the Brevig Mission RBD by the R35A mutation was confirmed via thermal shift assay up to concentrations of 400 μM (Figure S31). We show here that while the RBD alone has only a weak affinity for FAM-dsRNA (20,700 ± 2,400 nM, Figure S1), the full-length mutant binds significantly better at 210 ± 23 nM (Figure S2). It is evident that the presence of the full-length protein greatly stabilizes dsRNA binding interactions. Retention of dsRNA binding capabilities despite disruption of the RBD dimer interface has never before been reported, and so it is likely that this is a strain-specific interaction.

To ensure that the GST tag was not responsible for the observed binding, a RBD construct lacking the GST tag was created (His-RBD R35A). Evaluation of this construct resulted in a binding affinity of 51,000 ± 11,000 nM (Figure S3), indicating that the tag is not directly involved in binding. As was observed for the WT RBD constructs, these results indicate that while the GST tag is not responsible for dsRNA binding, it does stabilize complex formation.

Given that dsRNA binding was observed despite disruption of RBD dimerization, it was also evaluated whether the commonly mutated R38 and K41 residues were essential for dsRNA binding. R38A and K41A mutations are commonly included full-length NS1A to improve solubility of the protein, and have previously been seen to abolish binding of NS1A from other influenza strains to dsRNA.^15,29,32,33,36^ To accomplish this, double alanine mutants for both the RBD only (1–85) and full-length (1–230) constructs (His-GST-RBD R38A/K41A and His-GST-NS1A R38A/K41A) were created. Our data revealed that the Brevig Mission RBD R38A/K41A binds FAM-dsRNA with an affinity of 14,500 ± 1700 nM (Figure S4), while the full-length mutant has an affinity of 450 ± 89 nM (Figure S5).

All previous literature indicates that the R38A mutation alone should render the protein defunct.^13,29,32,33,36−38^ The only documented instance of R38A/K41A mutants binding to dsRNA occurred while using specially engineered aptamers.^39^ In this study, two strains, A/Hong Kong/213/03 (H5N1) and A/turkey/Italy/977/1999 (H7N1) were examined with one of the aptamers, both in their WT and R38A/K41A mutated forms. The mutated H7N1 strain exhibited very weak binding, with an affinity of approximately 50,000 nM, whereas the mutated H5N1 strain showed a binding affinity of 400 nM, as compared to subnanomolar affinities for the WT counterparts.^39^ Brevig Mission and A/Hong Kong/213/03 (H5N1) share four residues, R21, F22, T56, and R59, that are absent in the A/turkey/Italy/977/1999 (H7N1) strain; in fact, these strains exhibit complete sequence identity up to residue 70 where the linker region begins. This similarity in sequence and enhanced dsRNA binding lends credence to our hypothesis of strain-specific binding interactions.

Although the binding activity of the RBD to FAM-dsRNA is significantly weakened after introducing R35A or R38A/K41A mutations, it is not entirely eliminated. This suggests that other residues, such as F22, D53, T56, and R59, contribute to the enhanced activity in a cumulative fashion. Furthermore, our results highlight the need for continued evaluation of strain specific functions of NS1A; Full-length (1–230) Brevig Mission constructs containing R35A or R38A/K41A mutations retain significant ability to bind dsRNA, comparable to that of the WT Udorn NS1A (1–215).^33,36^ This may have significant implications for the design and application of anti-influenza therapeutics targeting NS1A.

### F22, D53, T56, and R59 in RBD Contribute to Increased Binding Capabilities of Brevig Mission NS1A

In order to elucidate the residues or regions responsible for the increased binding capabilities of the Brevig Mission RBD, a series of mutants in which particular individual residues were mutated to be identical to those found in the more commonly studied Udorn strain were designed (Figure 3). Residues that were anticipated to exert the most significant influence on RNA binding such as charged residues were mutated, while leaving untouched those that were highly conserved (e.g., glycine to alanine). Ultimately, this resulted in a library of five new constructs: R21Q, F22V, D53N, T56A, and R59H. While R46 and S42 have been shown to be important for dsRNA binding, we have excluded mutagenesis of these residues from the current study, as they are highly conserved between strains.^32^

**Figure 4:**
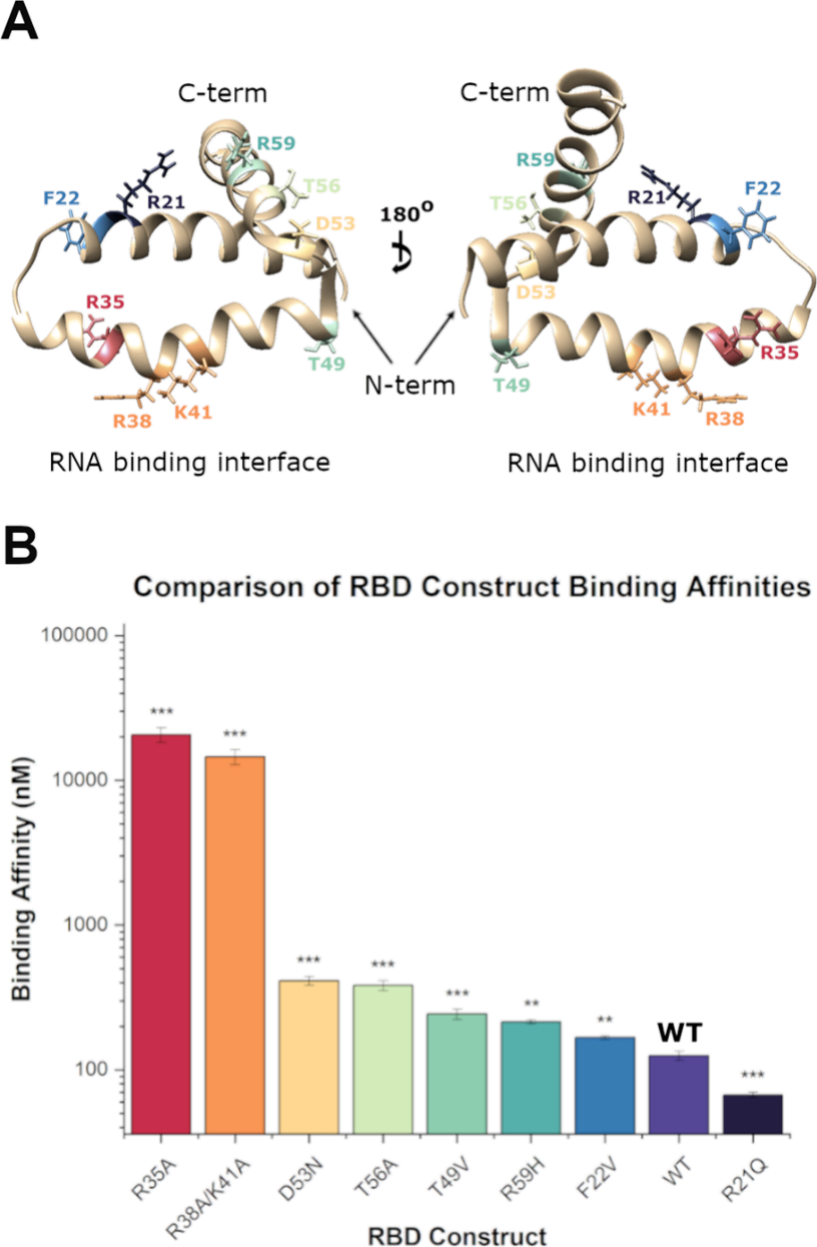
(A) Crystal structure of RBD monomer highlighting mutated residues. (B) Comparison of dsRNA binding affinity of RBD mutants as compared with the His-GST-RBD WT; analyzed via two-tailed student-*t* test. Error bars represent SD of triplicate runs. * = *p* < 0.05, ** = *p* < 0.01, *** = *p* < 0.001.

Of these five mutants, only His-GST-RBD R21Q resulted in an increased binding affinity for FAM-dsRNA (62 ± 3 nM, 2-fold vs His-GST-RBD). A salt bridge involving R21 and E72 on opposing chains has been observed in the Brevig Mission strain, which may result in structural deviations and subsequently, decreased dsRNA binding abilities.^40^ Conversely, F22V, D53N, T56A, and R59H all resulted in decreased dsRNA binding upon mutation (Table 1, Figure 4). Mutation of F22V resulted in a binding affinity of 170 ± 5 nM. This mutation has been found to induce a minor alteration in the loop region between the α-1 and α-2 helices, likely due to the bulky nature of the substituted residue.^15^ This slight structural change may lead to a less favorable conformation of the RBD upon dsRNA binding, resulting in the slightly reduced affinity.

**Table 1:** Averaged Triplicate Affinities and Standard Deviations of All RBD and Full-Length Constructs^a^

Construct	Binding affinity (nM, from FP)	Fold difference	P score
His-RBD	83 ± 11; 2,160 ± 460	-	-
His-RBD R35A	51,000 ± 11,000	158	0.0013
His-RBD T49V	3,300 ± 910	40	0.0049
His-GST-RBD	125 ± 9	-	-
His-GST-RBD R35A	20,700 ± 2,400	165	0.0001
His-GST-RBD R38A/K41A	14,500 ± 1,700	116	0.0001
His-GST-RBD T49V	240 ± 20	1.9	0.0007
His-GST-RBD R21Q	62 ± 3	0.5	0.0003
His-GST-RBD F22V	170 ± 5	1.3	0.0021
His-GST-RBD D53N	410 ± 28	3.3	0.0001
His-GST-RBD T56A	380 ± 29	3.1	0.0001
His-GST-RBD R59H	210 ± 17	2.0	0.0013
His-GST-NS1A R35A	210 ± 23	-	-
His-GST-NS1A R38A/K41A	450 ± 89	-	-
His-GST-NS1A W187A	124 ± 9	-	-

a Fold difference and P score calculated in comparison to similarly tagged WT construct. Statistical analysis was conducted via a two-tailed student t-test. Purification gels for these constructs shown in Figure S29.

### Stoichiometry of dsRNA Binding by Brevig Mission NS1A

Based on the existing models of NS1A interaction with dsRNA, it is known that multiple dimer units of NS1A bind to RNA in a tube-like structure to enhance sequestration.^15,16,18^ In most cases of the RBD alone, the binding stoichiometry of RBD dimer to dsRNA was found to be 1:1.^31,32^ However, when the dsRNA binding of A/Hong Kong/213/03 RBD was assessed against short aptamer RNAs, oligomerization of the RBD on the RNA was observed.^39,41^ Upon mutation of T49V this oligomerization was disrupted.^41^

The fEMSA assays shown above provided additional characterization of the nature of the interaction between the NS1 RBD and dsRNA (Figure 2B, 2C, and SI). Specifically, higher shifted bands were observed indicating that the RBD forms multiple, distinct complexes with FAM-dsRNA. While an exact quantification of this stoichiometry is not possible within the scope of this work, the highest band in Figure 2B is estimated to be indicative of a 3:1 binding stoichiometry of His-GST-RBD dimer to FAM-dsRNA. This estimation is supported by fEMSA analysis of a third RBD construct, GST-RBD, which shows evidence of two distinct complexes within the gel matrix, as well as a third complex within the well (Figure S27). This estimation of stoichiometry was made considering the current model of cooperative NS1A binding. In this model, three protein dimer units can envelop the dsRNA perimeter while also forming chains along the length.^15^ Given the short length of FAM-dsRNA, it is probable that any oligomerization would be restricted to three RBD dimer units encircling the dsRNA. Presumably, this mode of binding down the length of the RNA would be inhibited by the positioning of our N-terminal GST tag.

Considering the proposed stoichiometric ratios of His-GST-RBD alone (Figure 2B), it was hypothesized that introduction of the aforementioned T49V mutation in our construct would have the same effect. To assess this, two additional constructs were created: His-GST-RBD T49V and His-RBD T49V. When the His-GST-tagged T49V mutant was assessed, a binding affinity of 240 ± 20 nM was obtained, a 2-fold reduction from the WT, while the fEMSA was consistent with the presence of a 3:1 stoichiometric ratio of dimeric RBD to FAM-dsRNA (Table 1, Figure S6). The His-RBD T49V mutant yielded a much weaker affinity of 3,300 ± 910 nM, a 40-fold reduction from its similarly tagged WT (Table 1, Figure S7). The fEMSA for this construct retains the ability to form a multimeric complex, however the streaky nature of the bands indicate that it is much less stable than its GST-tagged counterpart. While the GST tag does appear to significantly stabilize this mutant, oligomerization of the RBD to the dsRNA is not abolished. This may be possible in part due to the previously discussed residues that contribute to the enhanced binding of the Brevig Mission dsRNA: F22, D53, T56, and R59.

### Brevig Mission NS1A ED Is Capable of Direct dsRNA Binding

In order to evaluate whether the ED was capable of direct interaction with dsRNA, a His-GST-ED construct (residues 86–230) was created. Evaluation of this construct resulted in a binding affinity of 1,900 ± 250 nM (Figure S13). As seen previously with the RBD, the fEMSA data is consistent with our previous estimation that the stoichiometry of the ED:dsRNA interaction is 3:1. Although the dsRNA affinity is ∼10-fold weaker than those observed for the His-GST-RBD, these results are in stark contrast to the literature, in which no direct interaction between the NS1A ED and RNA have been reported.^34^ Given that this appeared to be a novel function of the ED, characterization of its mechanism of binding was investigated.

### Dimerization of the Brevig Mission ED Is Not Essential for Direct dsRNA Binding

After establishing that the Brevig Mission ED does in fact directly interact with dsRNA, the next question to answer was whether ED dimerization is necessary. In order to form the tube-like structure that enhances sequestration of dsRNA, oligomerization of the full-length NS1A through the Trp-187 mediated ED dimer interface is necessary.^15,16,18^ When this tryptophan residue is mutated to an arginine in the Udorn NS1A protein, the affinity for dsRNA was reduced by 2-fold as compared to the full-length wild type;^18^ it has been observed that mutation to alanine results in even less efficient dsRNA binding.^37^ This assessment for the Brevig Mission ED was achieved through the use of both ED only (85–230) and full length (1–230) W187A mutants. The His-GST-ED W187A construct yielded a binding affinity of 2,500 ± 400 nM (Figure S14) which is not a significant change as compared to the WT ED, while the full-length His-GST-NS1A W187A had an affinity identical to that of the His-GST-RBD (124 ± 9 nM, Table 1 and Figure S15). Because the affinity of homodimerization of the ED is reported to range from 12 to 90 μM, it is understandable that with a low micromolar affinity for dsRNA that dimerization would not play a role in direct binding for the ED only construct.^18,19^ Our results for the full-length NS1A W187A construct fit well with the current literature reports which indicate that mutation of this residue disrupts higher-order oligomer formation, resulting in identical binding to dsRNA as the RBD only.

### Brevig Mission ED Binds to dsRNA Primarily through Its C-Terminal Tail

In order to elucidate the residues or regions responsible for the increased binding capabilities of the Brevig Mission ED, a series of mutants were created in which particular residues were mutated to be identical to those found in the more commonly studied Udorn strain. This resulted in a library of eight new constructs: M98L, S114P, M119I, D171I, E196K, R204G, P215T, and K227R.

Mutation of M98L or S114P did not result in a significant impact on dsRNA binding, resulting in affinities of 2,250 ± 170 nM and 2,260 ± 280 nM, respectively. In contrast, mutation of M119I resulted in a significantly reduced affinity of 3,900 ± 580 nM. Structural distinctions between the Brevig Mission and Udorn ED have been observed, particularly in the CPSF-30 binding region, despite only two mutational discrepancies in this area – A112E and M119I.^23^ Shen et al. suggest that one or both of these mutations could lead to altered binding properties to CPSF-30. Indeed, chemical shift perturbations were observed in M119 via NMR when small molecule mimics of the F2F3 fragment of CPSF-30 were introduced.^42^ Our findings support the hypothesis that M119 influences NS1A structure and, consequently, its binding interactions.

**Figure 5:**
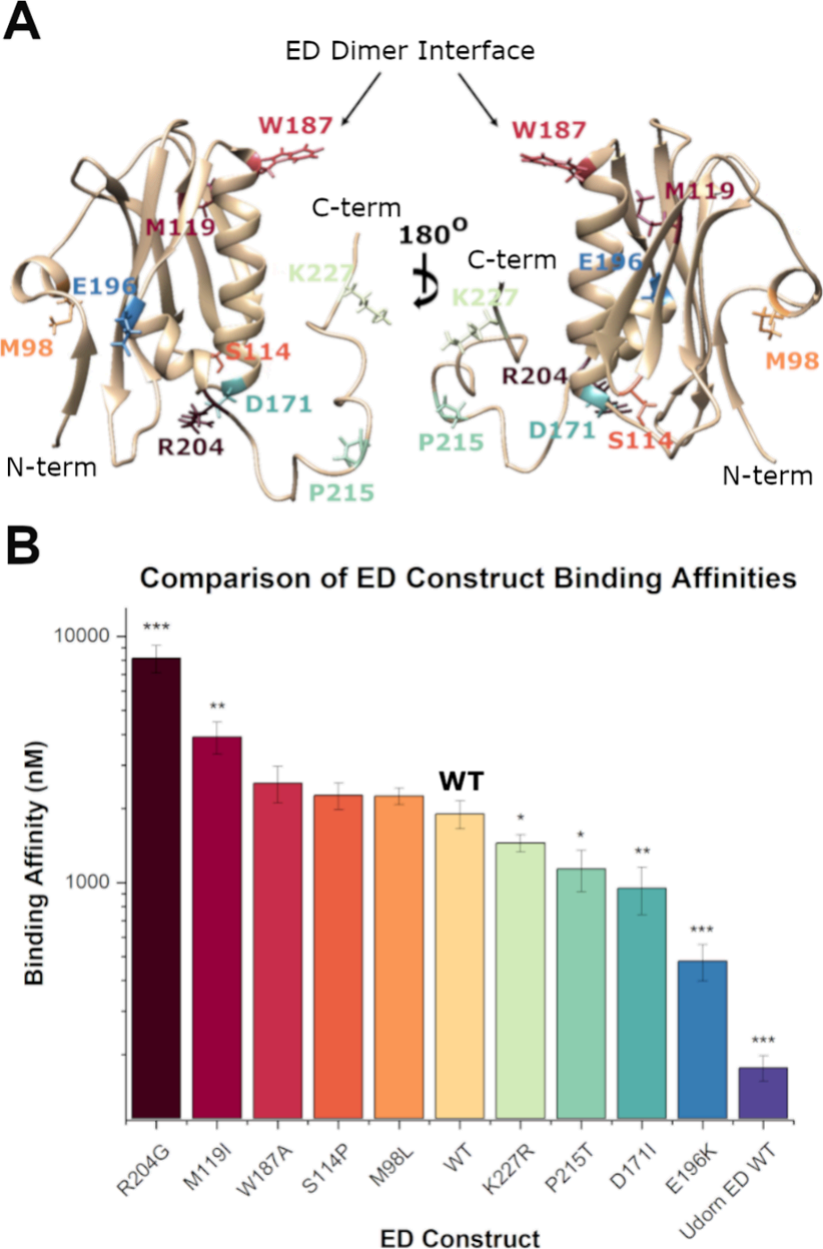
(A) Crystal structure of ED monomer highlighting mutated residues. (B) Comparison of dsRNA binding affinity of ED constructs as compared with the His-GST-ED WT; analyzed via two-tailed student-*t* test. Error bars represent SD of triplicate runs. * = *p* < 0.05, ** = *p* < 0.01, *** = *p* < 0.001.

The mutation which resulted in the greatest decrease in dsRNA binding was R204G. This construct yielded an affinity of 8,200 ± 1,000 nM, an approximate 4-fold decrease as compared to the WT ED. R204 is located on the short α-3 helix of the ED, indicating that residues in this region are involved in the binding of the ED to dsRNA. This hypothesis was supported by our assessment of E196K, P215T, and K227R, all of which resulted in increased binding to dsRNA (Table 2, Figure 5). Mutation of D171I also resulted in an increased affinity for dsRNA; however, the hydrophobic nature of this residue indicates that its role is likely not direct. We hypothesized that D171 interacts electrostatically with nearby R204, which decreases the ability of R204 to interact directly with dsRNA. Indeed, D171 was previously observed to interact with R204 and R140 via coordination with water in a crystal structure of the Brevig Mission ED.^43^ When a D171A mutant was crystallized, this was observed to result in disruption of the electrostatic interaction and altered flexibility of the nearby loop regions, which had subsequent effects on host protein binding.^43^

**Table 2:** Average Triplicate Affinities and Standard Deviations of All ED Constructs^a^

Construct	Binding affinity (nM, from FP)	Fold difference	P score
His-GST-ED	1,900 ± 250	-	-
His-GST-ED M98L	2,250 ± 170	1.2	0.1163
His-GST-ED S114P	2,260 ± 280	1.2	0.1657
His-GST-ED M119I	3,900 ± 580	2.1	0.0053
His-GST-ED D171I	950 ± 210	0.5	0.0070
His-GST-ED W187A	2,500 ± 400	1.3	0.0905
His-GST-ED E196K	480 ± 81	0.25	0.0007
His-GST-ED R204G	8,200 ± 1,000	4.3	0.0005
His-GST-ED P215T	1,140 ± 220	0.6	0.0160
His-GST-ED K227R	1,450 ± 120	0.76	0.0464
His-GST-ED Δ216–230	>100,000	>100	-
Udorn His-GST-ED	180 ± 21	-	-
Udorn His-GST-ED Δ216–237	>100,000	>1,000	-

a Fold difference and P score calculated in comparison to similarly tagged WT construct. Statistical analysis was conducted via a two-tailed student t-test. Purification gels for these constructs shown in Figure S30.

Because the residues which had the greatest impact on dsRNA binding were primarily located within the C-terminal tail region, a His-GST-ED Δ216–230 construct was designed to test the segment of the C-terminal tail that has been generally excluded in order to increase protein solubility.^19^ This construct resulted in a dsRNA binding affinity of >100,000 nM (Figure S24); due to concentration limitations, saturation of this binding event was unable to be attained. These results combined indicate that the C-terminal tail of the ED is the primary driving force of its ability to interact directly with dsRNA. Given that the C-terminal tail region is typically excluded from both full-length and ED only constructs, it is likely that inclusion of this region also contributes to the impressive retention of dsRNA binding by the full-length Brevig Mission R35A and R38A/K41A presented in this work when compared to prior reports.

### Udorn NS1A ED Is Capable of Direct dsRNA Binding When the C-Terminal Tail Is Retained

Based on our results indicating that the C-terminal tail of the Brevig Mission ED interacts directly with dsRNA, and that certain mutations toward the sequence of the Udorn strain enhanced this binding, recapitulation of this data using the Udorn strain was attempted. To achieve this, a Udorn His-GST-ED WT construct was created, as well as a construct lacking the C-terminal 22 residues of the tail region (Udorn His-GST-ED Δ216–237). The Udorn ED was found to bind to dsRNA with an affinity of 180 ± 21 nM, 10-fold stronger than the Brevig Mission ED (Figure S25). Assessment of Udorn GST-ED Δ216–237 resulted in severe abrogation of the interaction, with an affinity >100,000 nM (Figure S26). These results indicate that not only is the C-terminal tail of the ED responsible for its direct interaction with dsRNA, but that these interactions are strain-specific. It is likely that this dsRNA binding activity of the ED was never observed previously due to the tendency to remove the disordered C-terminal tail residues in order to increase protein solubility.^19^

Our studies, in conjunction with literature, indicate that the low nanomolar affinity of Udorn NS1A (1–215) for dsRNA is due entirely to the contribution of the RBD. Given that we show that Udorn ED (86–237) has a significant binding affinity in the same range as Udorn NS1A (1–215), the dsRNA binding of the full-length Udorn NS1A (1–237) is likely greatly enhanced by the C-terminal tail. In contrast, while we were unable to obtain data for the full-length WT Brevig Mission NS1A (1–230), we do show that the Brevig Mission RBD binds dsRNA with a higher affinity than Udorn NS1A (1–215), and that the Brevig Mission NS1A (1–230) mutant constructs all retain significant dsRNA binding in the nanomolar range. Because Brevig Mission NS1A W187A (1–230) has a dsRNA binding affinity similar to that of the Brevig Mission RBD alone, we can infer that there is little contribution from the direct ED-dsRNA interaction in the Brevig Mission strain within the constraints of our system. However, because dsRNA binding is not the sole contributor to influenza virulence, we are unable to evaluate the direct effect of the C-terminal tail region at this time. Due to the exclusion of the C-terminal tail in a majority of current literature, the biological relevance of this region is not fully understood.

Because the field is still lacking important information regarding the C-terminal tail of NS1A as well as strain-dependent differences, complete understanding of NS1A function during viral replication is hindered. However, some insight may be gained by comparison of NS1A to the NS1 protein from influenza B (NS1B). Despite low sequence homology, the EDs of NS1A and NS1B adopt similar conformations.^34^ Notably, the ED of NS1B was reported to bind as a monomer to short dsRNA with an affinity of 130 nM through basic surface residues.^34^ This dsRNA binding activity was observed to be necessary for optimal viral replication.^34^ Taken together with our observation of strain specific dsRNA binding of the NS1A ED, it is possible that the C-terminal tail of the NS1A ED also plays a similar role in viral replication. Several post-translational modifications (PTMs) occur at the C-terminus of the ED, and it is probable that these modifications govern its functionality throughout viral infection.^44−50^ Apart from its capacity for PTMs and dsRNA binding, the tail region can also interact with other proteins.^51−55^ Hence, it is reasonable to assume that the functionality of the C-terminal tail changes throughout infection to support the diverse roles of NS1A in a strain-dependent manner.

Further investigation into the functions of this region, as well as the strain-specific functions of NS1A as a whole, are needed in order to effectively target this protein with new therapeutics. This need is further highlighted by the recent emergence of an H5N1 avian flu strain which has recently been transmitted from cows to humans.^56^ While the majority of currently circulating influenza strains are derived from the 2009 pandemic strain (H1N1pdm09) and lack the C-terminal tail region, this newly circulating H5N1 strain does not; recently deposited sequence data show that H5N1 contains a C-terminal tail sequence similar, but not identical, to that of Brevig Mission (A/California/134/2024, NCBI). This new strain emergence has the potential to give rise to reassorted variants which could also contain the C-terminal tail region, further impacting influenza virulence, pathogenicity, and subsequent treatment methods.

## Conclusion

We have demonstrated that the dsRNA binding of NS1A is strain-specific. The Brevig Mission RBD contains multiple charged residues that enhance binding to FAM-dsRNA as compared to the more commonly studied Udorn strain, which may have contributed to the increased pathogenicity of the 1918 flu pandemic. We also show that the ED of NS1A is capable of directly interacting with dsRNA through its highly basic C-terminal tail, a novel mode of binding observed in both the Brevig Mission and Udorn NS1As. Furthermore, this novel interaction was demonstrated to be a function of strain dependence, with the Udorn ED binding to dsRNA 10-fold tighter than the Brevig Mission ED. Our findings highlight the importance of investigation of the strain-specific functionality of NS1A, and provide a new paradigm for consideration of the functional contributions of the C-terminal tail region. Further investigation of these areas is necessary for greater comprehension of the role of NS1A during influenza infection.

## Experimental Section

### Plasmid Construction

The A/Brevig Mission/1/1918 and A/Udorn/307/1972 genes were purchased from GenScript and cloned into the bacterial expression vector pET-16b via Gibson Assembly, as were the individual effector and RNA binding domains. An N-terminal GST tag was inserted between the 10xHis-tag and the protein, also via Gibson assembly (gene containing GST tag gifted from the lab of Dr. Zhihong Wang). All mutant constructs were created using the Q5 Site-Directed Mutagenesis Kit. A complete list of primers can be found in the SI (Table S1).

### Protein Expression and Purification

*Escherichia coli* BL21(DE3) Codon Plus (gifted from the lab of Dr. Zhihong Wang) cells were transformed with the recombinant plasmid and grown at 37 °C to an OD_600_ of 0.6, at which point the cells were induced with 0.5 mM IPTG overnight at 25 °C. For full-length constructs only, the cultures were induced with 0.3 mM IPTG at an OD_600_ of 0.3 at 18 °C overnight. The cells were harvested via centrifugation at 6,000 rpm for 20 min and pellets were stored at −20 °C until further use. The pellet was resuspended with 20 mL of lysis buffer (50 mM NaH_2_PO_4_, 300 mM NaCl, 10 mM imidazole, pH 8.0) with 1X COmplete protease inhibitor (Roche) and lysed by sonication (10 s on, 60 s off, for a total sonication time of 4 min at 30% amplitude), followed by lysis with 7 mg/mL lysozyme (Sigma-Aldrich) and 1,500 units GENIUS nuclease (Santa Cruz Biotechnology) at 37 °C for 30 min. The cell debris was pelleted at 12,000 rpm for 45 min, and the clarified lysate was incubated with nickel-charged Profinity IMAC resin (Bio-Rad) for 1 h rotating at 4 °C. On a gravity column, the resin was then washed with 10 mM and 100 mM imidazole buffers (50 mM K_2_HPO_4_, 300 mM KCl, pH = 8), followed by a step-gradient elution with varying concentrations of imidazole (100 mM, 300 mM, 500 mM, 1 M). Fractions were assessed via SDS-PAGE with Coomassie staining (Figures S29 & S30). Only fractions with purity >95% by Coomassie staining were pooled, concentrated, and buffer exchanged into the final storage buffer (10% glycerol, 50 mM Tris, 1.3 mM EDTA, 100 mM NaCl, pH = 8) using Bio-Rad Econo-Pac 10-DG desalting columns and concentrated via centrifugation using Vivaspin 15R concentrators (Sartorius). Ni-NTA purifications which did not result in >95% pure protein were further purified using size exclusion chromatography (SEC) with the final storage buffer. For SEC purifications, a Bio-Rad ENrich SEC-70 or SEC-650 column was used in conjunction with a Bio-Rad NGC Chromatography System at a flow rate of 0.5 mL/min.

### Fluorescence Polarization Assays

Fluorescence polarization assays were carried out as previously described by Cho et al.^33^ FITC-labeled RNAs were obtained from IDT and resuspended in ddH_2_O to a concentration of 100 μM and stored at −80 °C until further use. Double stranded RNA was annealed by combining equal amounts of fluorescein-labeled sense and antisense RNA (FAM-CCAUCCUCUACAGGCG (sense) and FAM-CGCCUGUAGAGGAUGG (antisense)) in 1x hybridization buffer (50 mM Tris-HCl, 50 mM KCl, 0.02% Tween-20, pH 8.0) for 2 min at 90 °C and was allowed to cool to room temperature for 1 h. Fluorescence polarization assays were carried out in a Corning black low-volume round-bottom 384-well plates using a BioTek H1 Synergy plate reader. A final concentration of 10 nM dsRNA was selected as the optimal concentration based on initial FP independence of FI experiments. Each well of binding affinity experiments contained 10 nM dsRNA, 1x hybridization buffer, and the indicated amount of protein. Plates were allowed to incubate for 1 h at room temperature before reading. Data was fit to a logistic or biphasic curve in OriginPro 2021b to obtain binding affinity.

### fEMSA Assays

Samples from FP assays were directly mixed with Orange-G loading dye and analyzed further with fEMSA assays. Orange-G dye in 30% glycerol was used to assist in loading. 5% acrylamide native gels were prerun at 80 V for 1 h prior to sample addition, then run at 80 V for ∼1 h after sample addition. Gels were visualized using an Amersham Typhoon imager on the Cy2 setting (laser 488 nm). Fraction bound was calculated by quantifying free RNA in Image Studio Lite. Data was fit to a logistic curve in Origin 2021b.

## Supporting Information Available

FP and fEMSA data for all constructs, purification gels, thermal shift assay data, supplemental experimental methods, and a list of mutagenesis primers (PDF)

## Abbreviations

ED: effector domain
FP: fluorescence polarization
fEMSA: fluorescence-based electrophoretic mobility shift assay
GST: Glutathione S-transferase
NS1A: nonstructural protein 1 of influenza A
PTM: post-translational modification
RBD: RNA binding domain
