# Supplementary material for: Nonstructural Protein 1 of Influenza A (NS1A) Demonstrates Strain-Specific dsRNA Binding Capabilities
Source: ACS Infect Dis. 2025 Mar 13;11(4):859–68. doi: 10.1021/acsinfecdis.4c00882 (PMC11997982; doi:10.1021/acsinfecdis.4c00882)
Supplement: Supplementary file 1 — id4c00882_si_001.pdf [file id4c00882_si_001.pdf]

Supporting Information

Nonstructural protein 1 of influenza A (NS1A) demonstrates strain-specific dsRNA binding capabilities

Veronica A. Smith[1], Aubrey R. Schall[1], and John W. Tomsho[1*]

[1]Saint Joseph's University, Department of Chemistry & Biochemistry, University City Campus, 600 South 43rd Street, Philadelphia, PA 19104, USA

*Corresponding Author: jtomsho@sju.edu

This file contains:

41 Pages

32 Figures

Supplemental Information Experimental Methods

1 Table

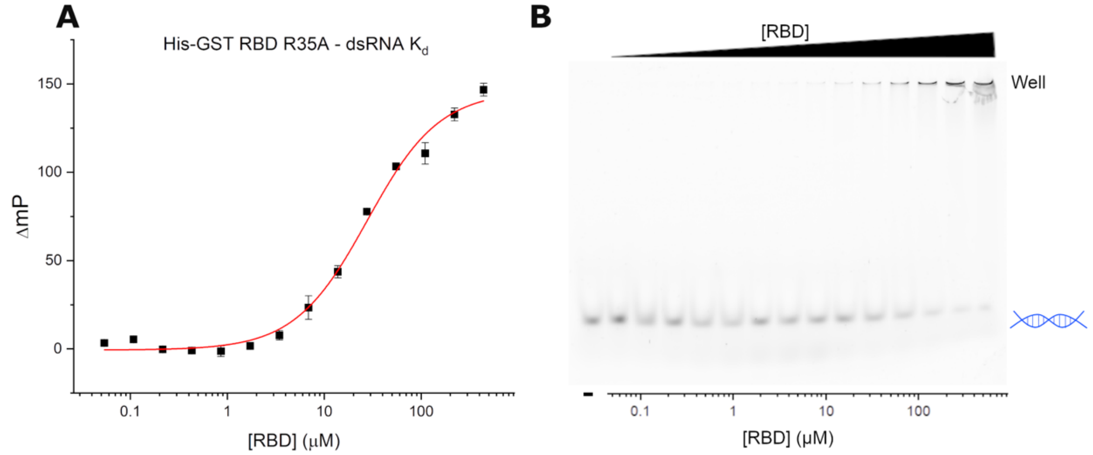

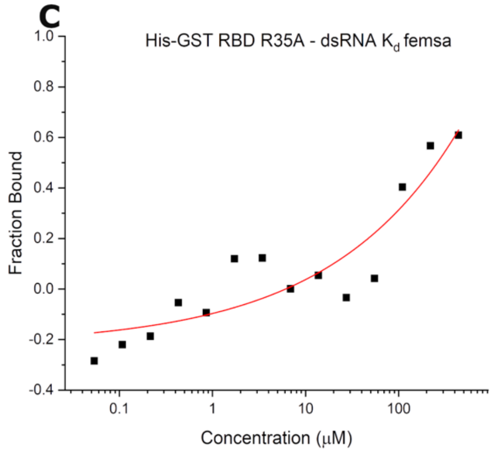

	FP	fEMSA
Average affinity (nM)	20,700 ± 2,400	>75,000
Average R^2	0.9909	N/A

Figure S1. Binding affinity of Brevig Mission His-GST-RBD R35A for dsRNA. A&B) Paired fluorescence polarization data for binding affinity of His-GST-RBD R35A (K_d = 20,700 ± 2,400 nM) and fEMSA assay showing increased dsRNA binding with increasing His-GST-RBD R35A concentration (K_d >75,000 nM). Leftmost lane is RNA only. Free dsRNA is depicted by blue helix on the right, with RBD-dsRNA complexes shifted above as indicated. **C)** Fit of fEMSA data obtained by plotting free dsRNA from gel assay. Data shown are representative of three independent experiments while values are presented as averages with standard deviations. Affinity was not able to be accurately assessed by fEMSA due to lack of saturation.

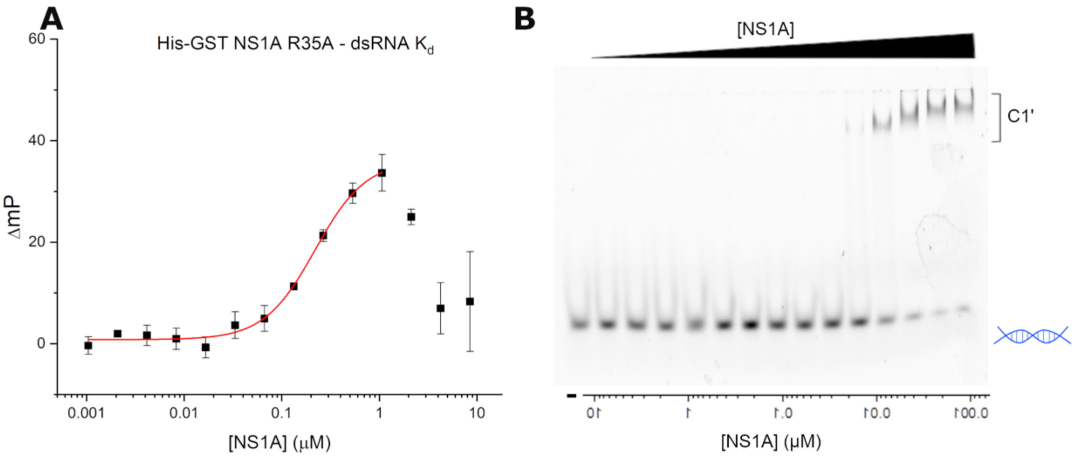

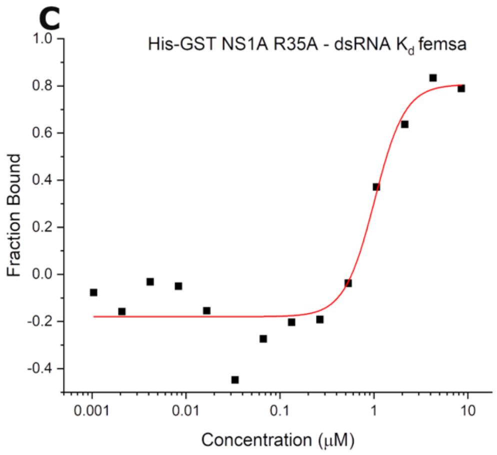

	FP	fEMSA
Average affinity (nM)	210 ± 23	860 ± 170
Average R^2	0.9836	0.90598

Figure S2. Binding affinity of Brevig Mission His-GST-NS1A R35A for dsRNA. A&B) Paired fluorescence polarization data for binding affinity of His-GST-NS1A R35A (K_d = 210 ± 23 nM) and fEMSA assay showing increased dsRNA binding with increasing His-GST-NS1A R35A concentration (K_d = 860 ± 170 nM). Leftmost lane is RNA only. Free dsRNA is depicted by blue helix on the right, with NS1A-dsRNA complexes shifted above as indicated. Highest three concentration data points in FP assay excluded from fit due to fluorescence quenching. **C)** Fit of fEMSA data obtained by plotting free dsRNA from gel assay. Data shown are representative of three independent experiments while values are presented as averages with standard deviations.

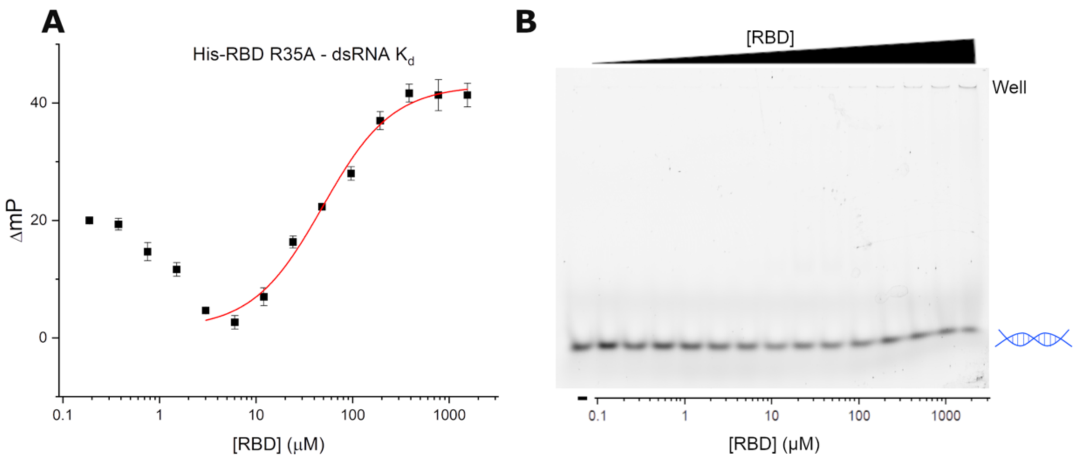

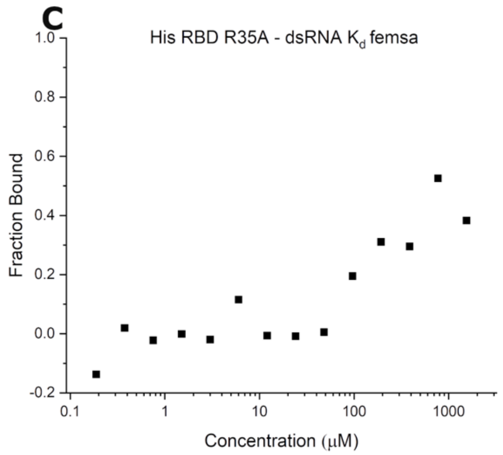

	**FP**	**fEMSA**
Average affinity (nM)	51,000 ± 11,000	>540,000
Average R^2	0.97852	N/A

Figure S3. Binding affinity of Brevig Mission His-RBD R35A for dsRNA. A&B) Paired fluorescence polarization data for binding affinity of His-RBD R35A (K_d = 51,000 ± 11,000 nM) and fEMSA assay showing increased dsRNA binding with increasing His-RBD R35A concentration (K_d >540,000 nM). Leftmost lane is RNA only. Free dsRNA is depicted by blue helix on the right, with RBD-dsRNA complexes shifted above as indicated. **C)** Fit of fEMSA data obtained by plotting free dsRNA from gel assay. Data shown are representative of three independent experiments while values are presented as averages with standard deviations. Affinity was not able to be accurately assessed by fEMSA due to lack of saturation.

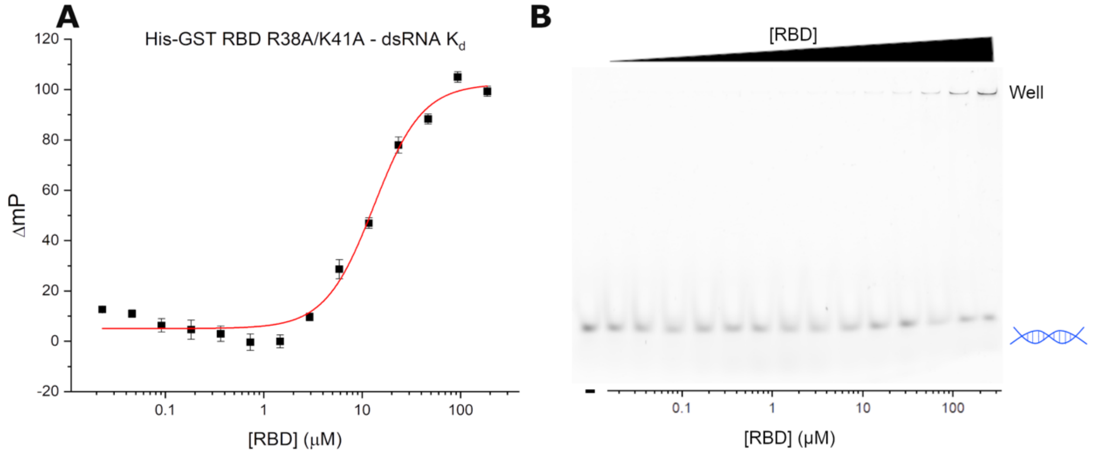

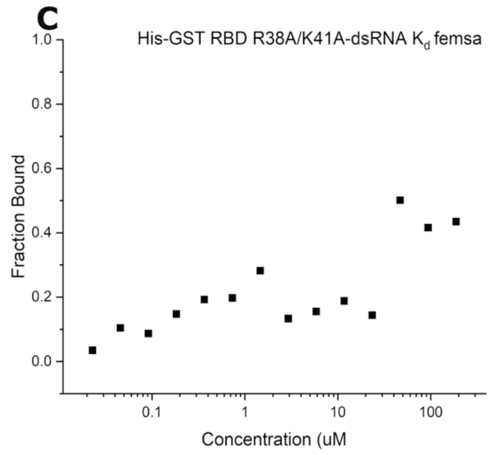

	FP	fEMSA
Average affinity (nM)	14,500 ± 1,700	N/A
Average R^2	0.98522	N/A

Figure S4. Binding affinity of Brevig Mission His-GST-RBD R38A/K41A for dsRNA. A&B) Paired fluorescence polarization data for binding affinity of His-GST-RBD R38A/K41A (K_d = 14,500 ± 1,700 nM) and fEMSA assay showing increased dsRNA binding with increasing His-GST-RBD R38A/K41A concentration (Affinity not obtained). Leftmost lane is RNA only. Free dsRNA is depicted by blue helix on the right, with RBD-dsRNA complexes shifted above as indicated. **C)** Fit of fEMSA data obtained by plotting free dsRNA from gel assay. Data shown are representative of three independent experiments while values are presented as averages with standard deviations. Affinity was not able to be accurately assessed by fEMSA due to lack of saturation.

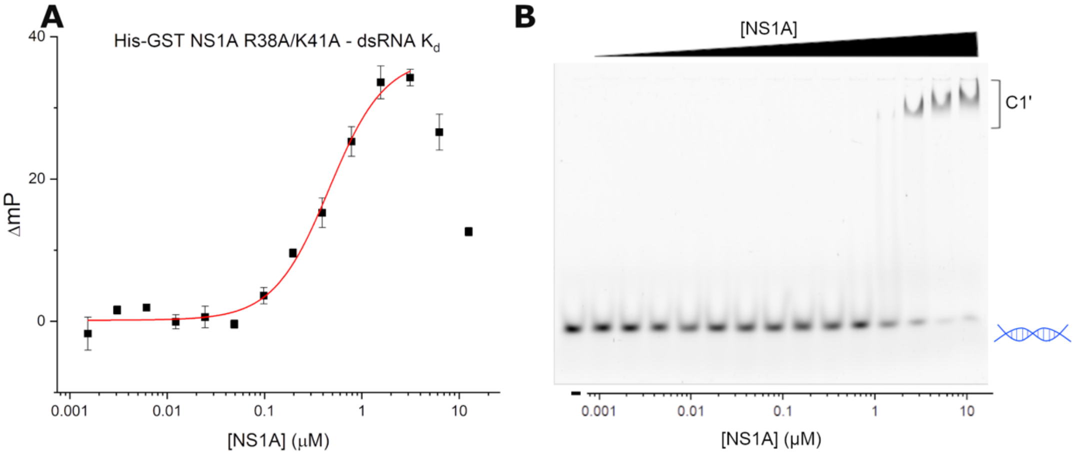

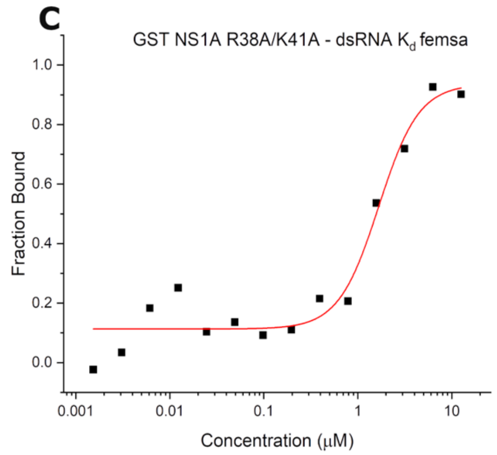

	FP	fEMSA
Average affinity (nM)	450 ± 89	1,530 ± 240
Average R^2	0.9844	0.92666

Figure S5. Binding affinity of Brevig Mission His-GST-NS1A R38A/K41A for dsRNA. A&B) Paired fluorescence polarization data for binding affinity of His-GST-NS1A R38A/K41A (K_d = 450 ± 89 nM) and fEMSA assay showing increased dsRNA binding with increasing His-GST-NS1A R38A/K41A concentration (K_d = 1,530 ± 240 nM). Leftmost lane is RNA only. Free dsRNA is depicted by blue helix on the right, with NS1A-dsRNA complexes shifted above as indicated. Highest two concentration data points in FP assay excluded from fit due to fluorescence quenching. **C)** Fit of fEMSA data obtained by plotting free dsRNA from gel assay. Data shown are representative of three independent experiments while values are presented as averages with standard deviations.

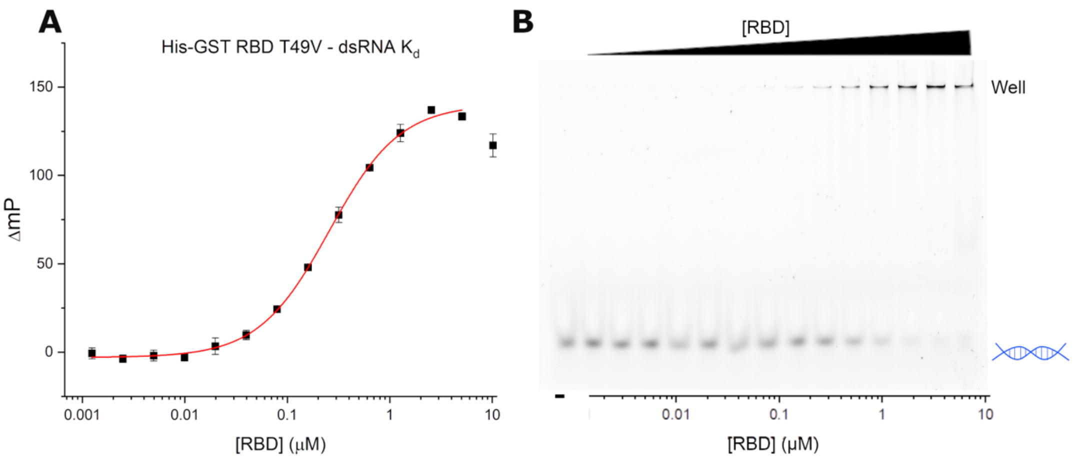

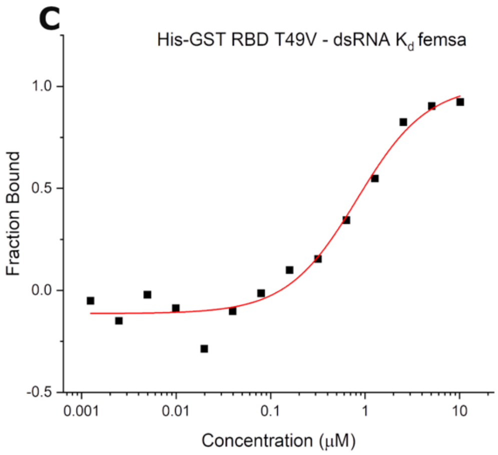

	**FP**	**fEMSA**
Average affinity (nM)	240 ± 20	1150 ± 320
Average R^2	0.9948	0.96630

Figure S6. Binding affinity of Brevig Mission His-GST-RBD T49V for dsRNA. A&B) Paired fluorescence polarization data for binding affinity of His-GST-RBD T49V (K_d = 240 ± 20 nM) and fEMSA assay showing increased dsRNA binding with increasing His-GST-RBD T49V concentration (K_d = 1150 ± 320 nM). Leftmost lane is RNA only. Free dsRNA is depicted by blue helix on the right, with RBD-dsRNA complexes shifted above. Highest concentration data point in FP assay excluded from fit due to fluorescence quenching. **C)** Fit of fEMSA data obtained by plotting free dsRNA from gel assay. Data shown are representative of three independent experiments for FP and two independent experiments for fEMSA, while values are presented as averages with standard deviations.

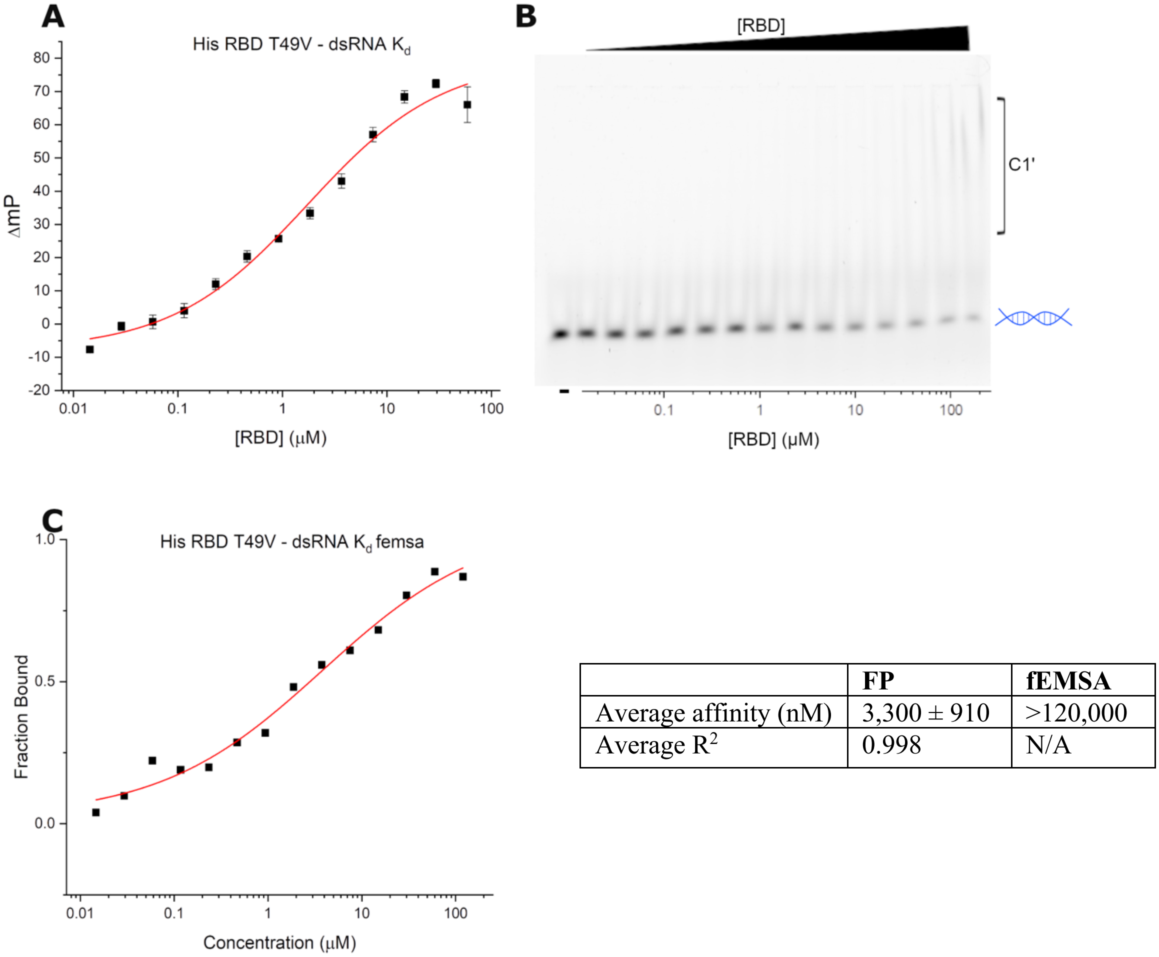

	FP	fEMSA
Average affinity (nM)	3,300 ± 910	>120,000
Average R^2	0.998	N/A

Figure S7. Binding affinity of Brevig Mission His-RBD T49V for dsRNA. A&B) Paired fluorescence polarization data for binding affinity of His-RBD T49V (K_d = 3,300 ± 910 nM) and fEMSA assay showing increased dsRNA binding with increasing His-RBD T49V concentration (K_d = >120,000 nM). Leftmost lane is RNA only. Free dsRNA is depicted by blue helix on the right, with RBD-dsRNA complexes shifted above as indicated. **C)** Fit of fEMSA data obtained by plotting free dsRNA from gel assay. Affinity not able to be accurately assessed due to lack of saturation. Data shown are representative of three independent experiments while values are presented as averages with standard deviations. Affinity was not able to be accurately assessed by fEMSA due to lack of saturation.

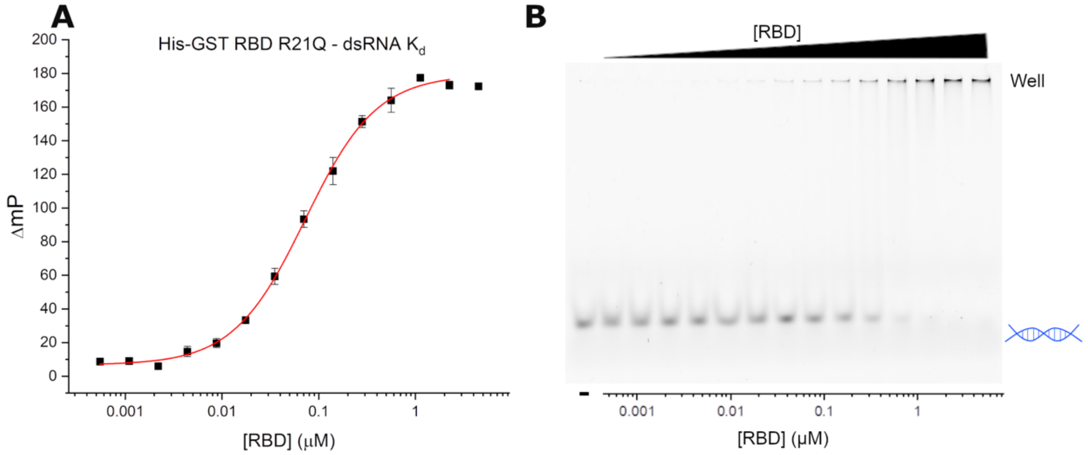

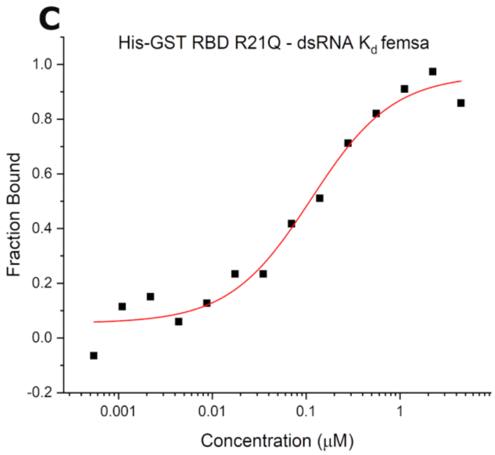

	FP	fEMSA
Average affinity (nM)	62 ± 3	145 ± 30
Average R^2	0.998	0.97162

Figure S8. Binding affinity of Brevig Mission His-RBD R21Q for dsRNA. A&B) Paired fluorescence polarization data for binding affinity of His-RBD R21Q (K_d = 67 ± 3 nM) and fEMSA assay showing increased dsRNA binding with increasing His-RBD R21Q concentration (K_d = 145 ± 30 nM). Leftmost lane is RNA only. Free dsRNA is depicted by blue helix on the right, with RBD-dsRNA complexes shifted above as indicated. **C)** Fit of fEMSA data obtained by plotting free dsRNA from gel assay. Data shown are representative of three independent experiments while values are presented as averages with standard deviations.

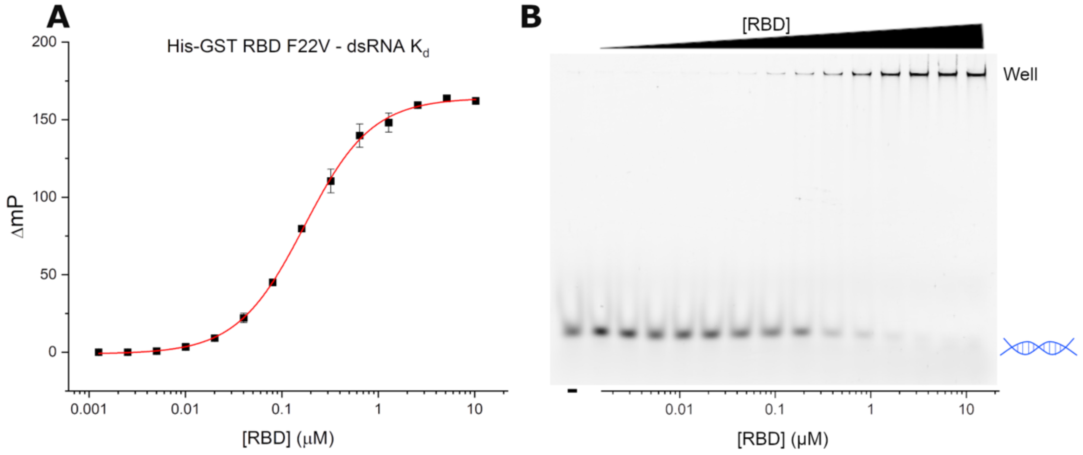

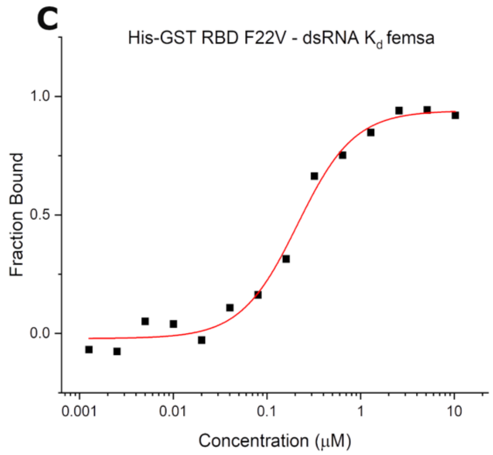

	FP	fEMSA
Average affinity (nM)	170 ± 5	230 ± 45
Average R^2	0.99908	0.97064

Figure S9. Binding affinity of Brevig Mission His-RBD F22V for dsRNA. A&B) Paired fluorescence polarization data for binding affinity of His-RBD F22V (K_d = 170 ± 5 nM) and fEMSA assay showing increased dsRNA binding with increasing His-RBD F22V concentration (K_d = 230 ± 45 nM). Leftmost lane is RNA only. Free dsRNA is depicted by blue helix on the right, with RBD-dsRNA complexes shifted above as indicated. **C)** Fit of fEMSA data obtained by plotting free dsRNA from gel assay. Data shown are representative of three independent experiments while values are presented as averages with standard deviations.

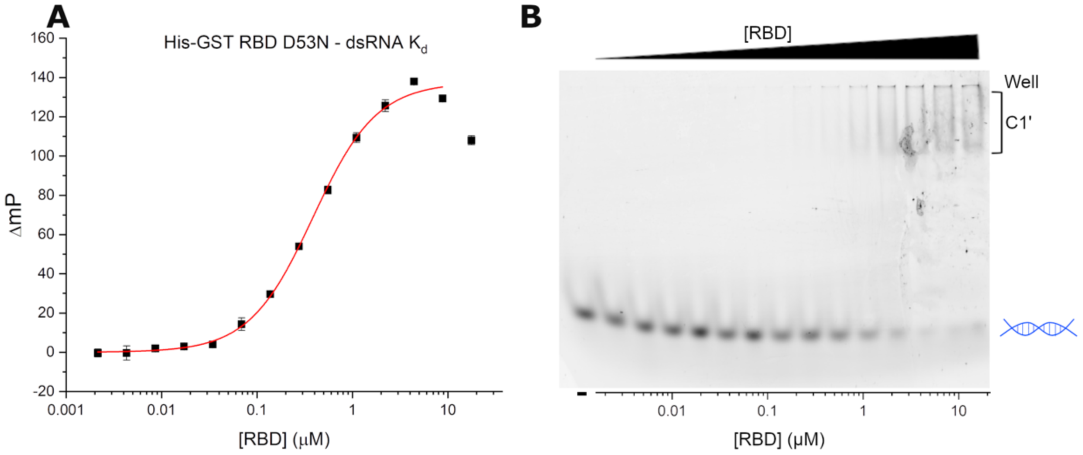

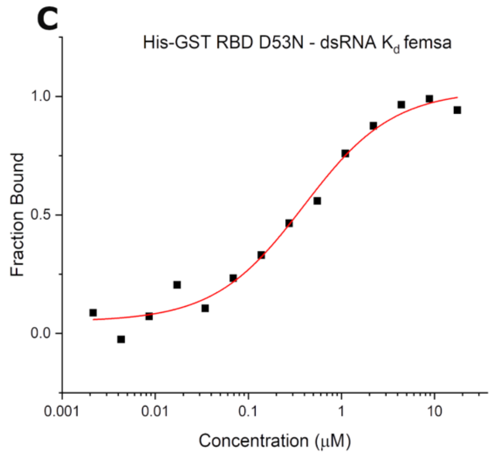

	FP	fEMSA
Average affinity (nM)	410 ± 28	710 ± 410
Average R^2	0.99580	0.92361

Figure S10. Binding affinity of Brevig Mission His-RBD D53N for dsRNA. A&B) Paired fluorescence polarization data for binding affinity of His-RBD D53N (K_d = 410 ± 28 nM) and fEMSA assay showing increased dsRNA binding with increasing His-RBD D53N concentration (K_d = 710 ± 410 nM). Leftmost lane is RNA only. Free dsRNA is depicted by blue helix on the right, with RBD-dsRNA complexes shifted above as indicated. Highest concentration data point in FP assay excluded from fit due to fluorescence quenching. **C)** Fit of fEMSA data obtained by plotting free dsRNA from gel assay. Data shown are representative of three independent experiments while values are presented as averages with standard deviations.

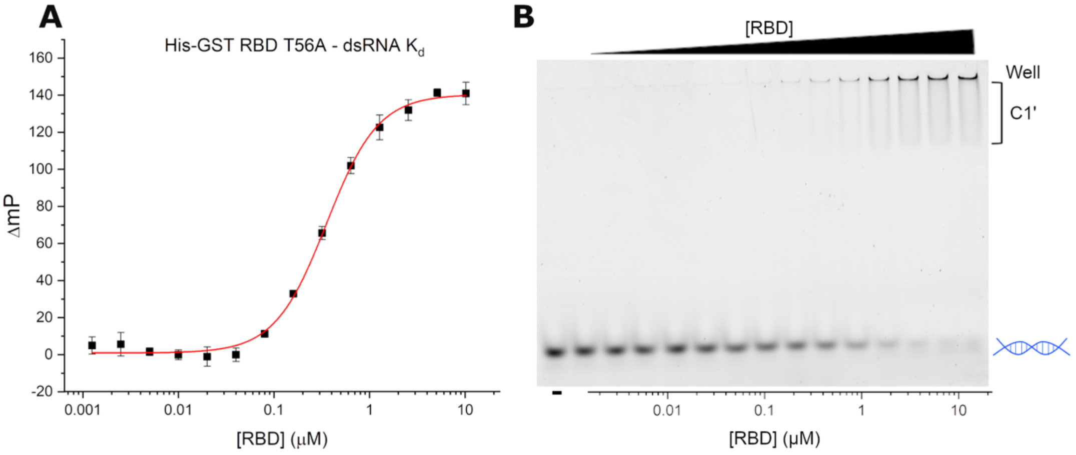

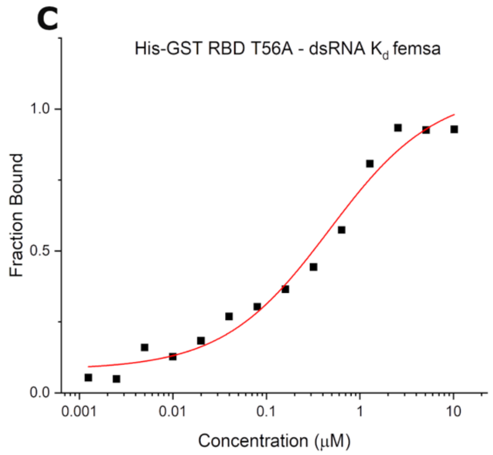

	FP	fEMSA
Average affinity (nM)	380 ± 29	780 ± 730
Average R^2	0.99601	0.97242

Figure S11. Binding affinity of Brevig Mission His-RBD T56A for dsRNA. A&B) Paired fluorescence polarization data for binding affinity of His-RBD T56A (K_d = 380 ± 29 nM) and fEMSA assay showing increased dsRNA binding with increasing His-RBD T56A concentration (K_d = 780 ± 730 nM). Leftmost lane is RNA only. Free dsRNA is depicted by blue helix on the right, with RBD-dsRNA complexes shifted above depicted as indicated. **C)** Fit of fEMSA data obtained by plotting free dsRNA from gel assay. Data shown are representative of three independent experiments while values are presented as averages with standard deviations.

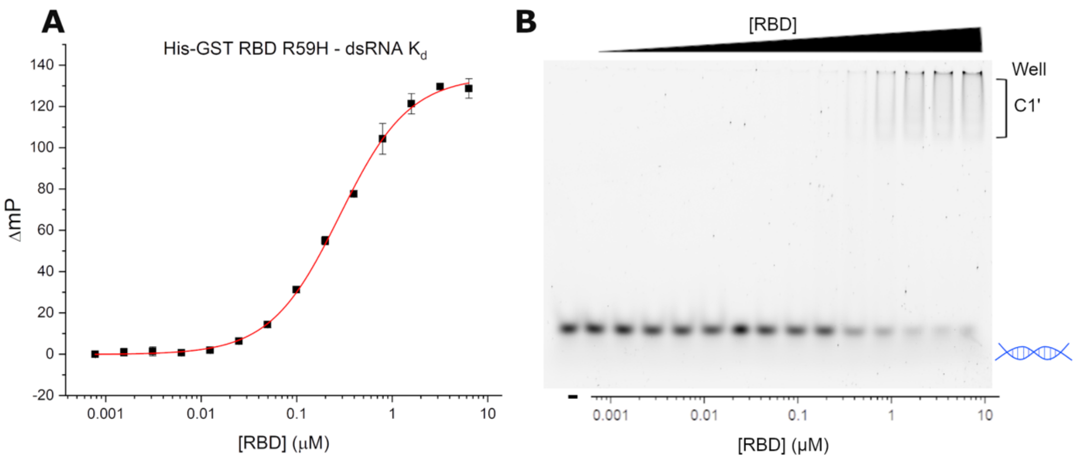

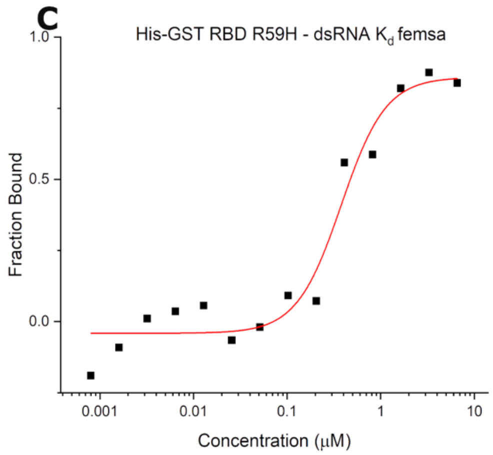

	FP	fEMSA
Average affinity (nM)	210 ± 17	580 ± 110
Average R^2	0.9935	0.9353

Figure S12. Binding affinity of Brevig Mission His-RBD R59H for dsRNA. A&B) Paired fluorescence polarization data for binding affinity of His-RBD R59H (K_d = 210 ± 17 nM) and fEMSA assay showing increased dsRNA binding with increasing His-RBD R59H concentration (K_d = 580 ± 110 nM). Leftmost lane is RNA only. Free dsRNA is depicted by blue helix on the right, with RBD-dsRNA complexes shifted above as indicated. **C)** Fit of fEMSA data obtained by plotting free dsRNA from gel assay. Data shown are representative of three independent experiments while values are presented as averages with standard deviations.

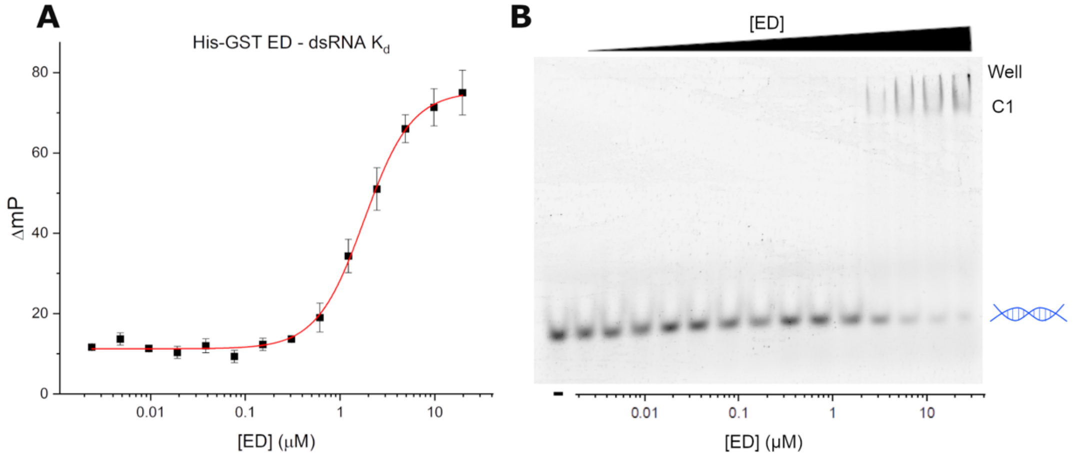

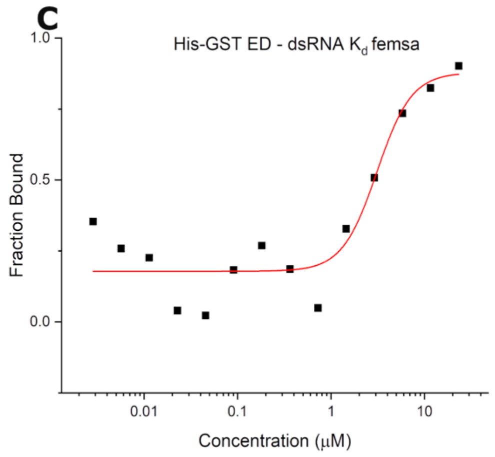

	FP	fEMSA
Average affinity (nM)	1900 ± 250	3,500 ± 800
Average R^2	0.97757	0.90135

Figure S13. Binding affinity of Brevig Mission His-GST-ED for dsRNA. A&B) Paired fluorescence polarization data for binding affinity of His-GST-ED (K_d = 1,900 ± 250 nM) and fEMSA assay showing increased dsRNA binding with increasing His-GST-ED concentration (K_d = 3,500 ± 800 nM). Leftmost lane is RNA only. Free dsRNA is depicted by blue helix on the right, with ED-dsRNA complexes shifted above as indicated. **C)** Fit of fEMSA data obtained by plotting free dsRNA from gel assay. Data shown are representative of three independent experiments while values are presented as averages with standard deviations.

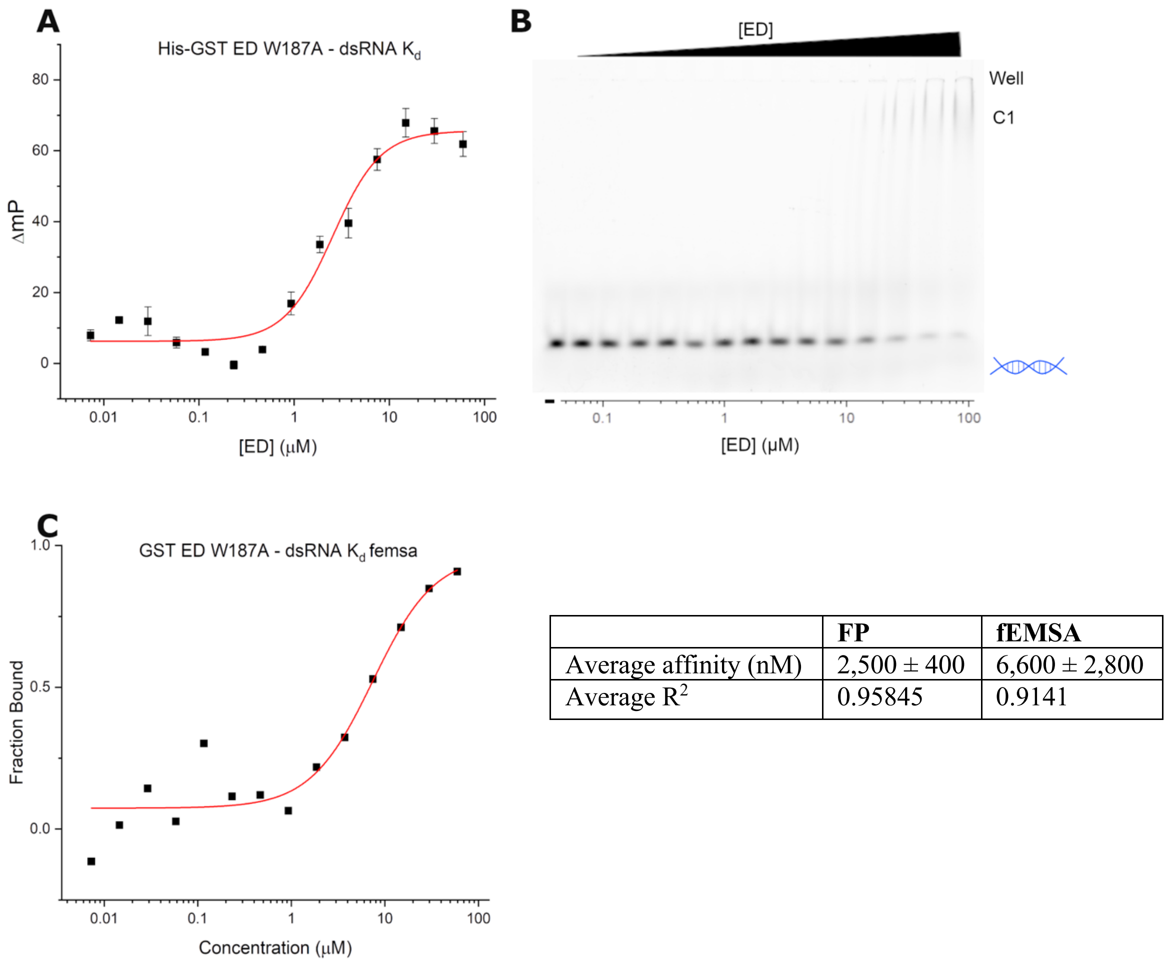

	FP	fEMSA
Average affinity (nM)	2,500 ± 400	6,600 ± 2,800
Average R^2	0.95845	0.9141

Figure S14. Binding affinity of Brevig Mission His-GST-ED W187A for dsRNA. A&B) Paired fluorescence polarization data for binding affinity of His-GST-ED W187A (K_d = 2,500 ± 400 nM) and fEMSA assay showing increased dsRNA binding with increasing His-GST-ED W187A concentration (K_d = 6,600 ± 2,800 nM). Leftmost lane is RNA only. Free dsRNA is depicted by blue helix on the right, with ED-dsRNA complexes shifted above as indicated. **C)** Fit of fEMSA data obtained by plotting free dsRNA from gel assay. Data shown are representative of three independent experiments for FP and two independent experiments for fEMSA, while values are presented as averages with standard deviations.

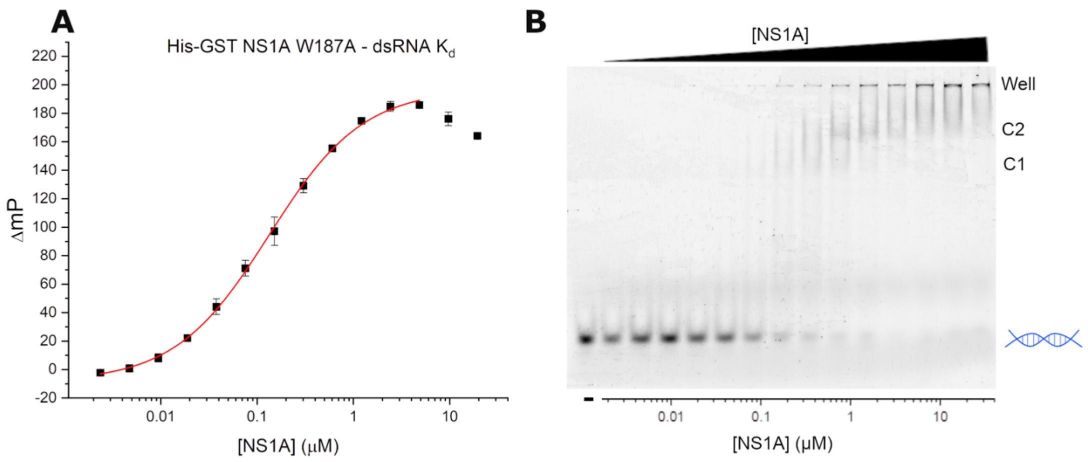

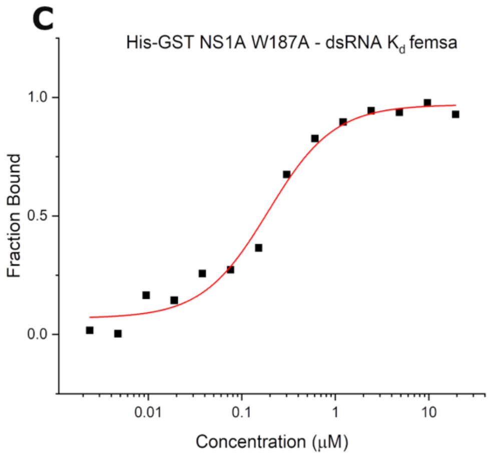

	FP	fEMSA
Average affinity (nM)	124 ± 8	130 ± 27
Average R^2	0.9987	0.96871

Figure S15. Binding affinity of Brevig Mission His-GST-NS1A W187A for dsRNA. A&B) Paired fluorescence polarization data for binding affinity of His-GST-NS1A W187A (K_d = 124 ± 8 nM) and fEMSA assay showing increased dsRNA binding with increasing His-GST-NS1A W187A concentration (K_d = 130 ± 27 nM). Leftmost lane is RNA only. Free dsRNA is depicted by blue helix on the right, with NS1A-dsRNA complexes shifted above as indicated. Highest two concentration data points in FP assay excluded from fit due to fluorescence quenching. **C)** Fit of fEMSA data obtained by plotting free dsRNA from gel assay. Data shown are representative of three independent experiments while values are presented as averages with standard deviations.

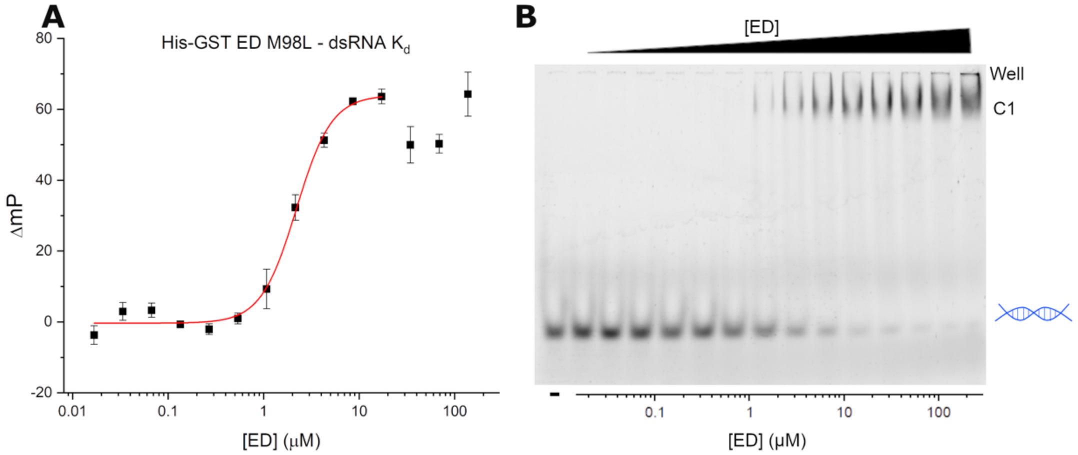

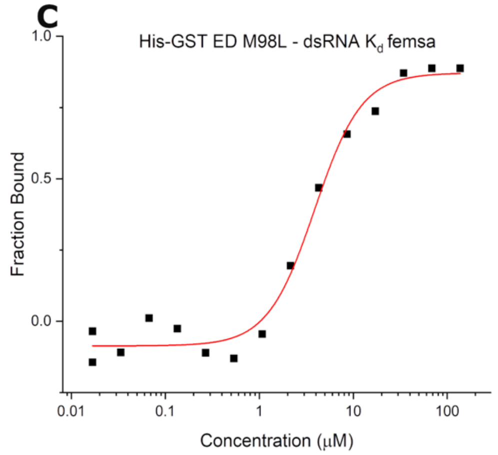

	FP	fEMSA
Average affinity (nM)	2,250 ± 170	3,020 ± 400
Average R^2	0.98696	0.98256

Figure S16. Binding affinity of Brevig Mission His-GST-ED M98L for dsRNA. A&B) Paired fluorescence polarization data for binding affinity of His-GST-ED M98L (K_d = 2,250 ± 170 nM) and fEMSA assay showing increased dsRNA binding with increasing His-GST-ED M98L concentration (K_d = 3,020 ± 400 nM). Leftmost lane is RNA only. Free dsRNA is depicted by blue helix on the right, with ED-dsRNA complexes shifted above as indicated. Highest three concentration data points in FP assay excluded from fit due to observed protein precipitation. **C)** Fit of fEMSA data obtained by plotting free dsRNA from gel assay. Data shown are representative of three independent experiments while values are presented as averages with standard deviations.

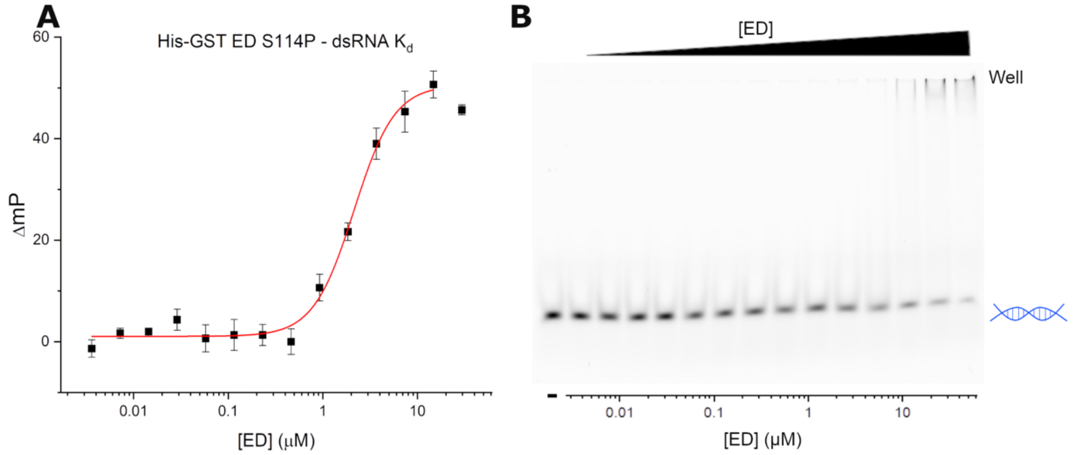

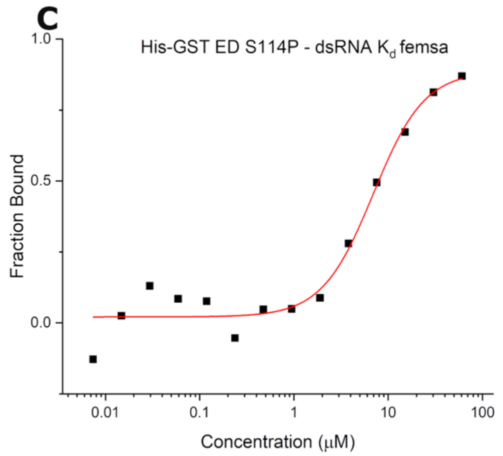

	FP	fEMSA
Average affinity (nM)	2,260 ± 280	6,200 ± 2,300
Average R^2	0.9813	0.86947

Figure S17. Binding affinity of Brevig Mission His-GST-ED S114P for dsRNA. A&B) Paired fluorescence polarization data for binding affinity of His-GST-ED S114P (K_d = 2,260 ± 280 nM) and fEMSA assay showing increased dsRNA binding with increasing His-GST-ED S114P concentration (K_d = 6,200 ± 2,300 nM). Leftmost lane is RNA only. Free dsRNA is depicted by blue helix on the right, with ED-dsRNA complexes shifted above as indicated. Highest concentration data point in FP assay excluded from fit due to fluorescence quenching. **C)** Fit of fEMSA data obtained by plotting free dsRNA from gel assay. Data shown are representative of three independent experiments while values are presented as averages with standard deviations.

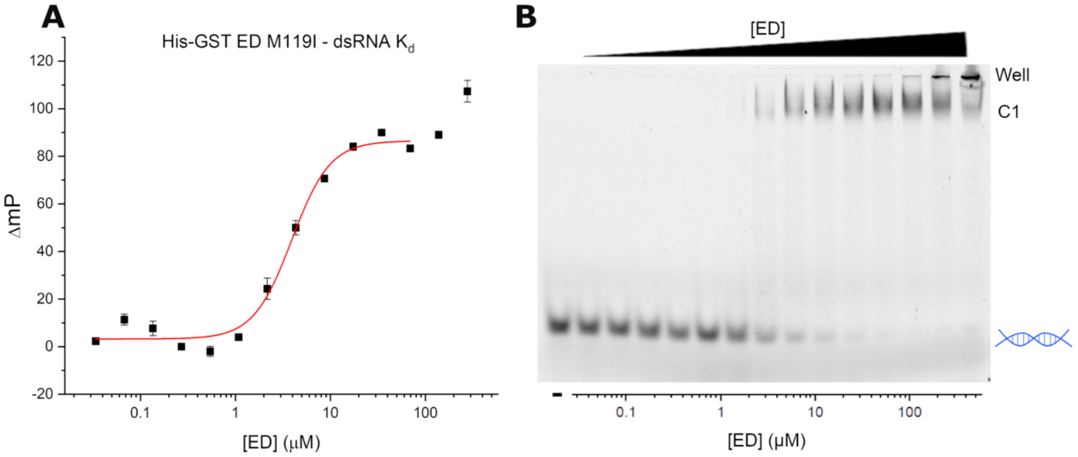

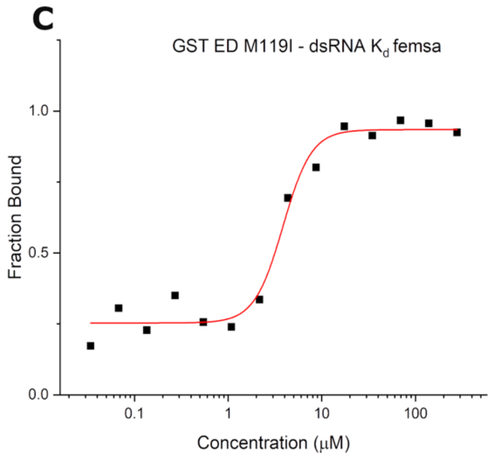

	FP	fEMSA
Average affinity (nM)	3,900 ± 580	2,810 ± 350
Average R^2	0.96197	0.9765

Figure S18. Binding affinity of Brevig Mission His-GST-ED M119I for dsRNA. A&B) Paired fluorescence polarization data for binding affinity of His-GST-ED M119I (K_d = 3,900 ± 580 nM) and fEMSA assay showing increased dsRNA binding with increasing His-GST-ED M119I concentration (K_d = 2,810 ± 350 nM). Leftmost lane is RNA only. Free dsRNA is depicted by blue helix on the right, with-ED-dsRNA complexes shifted above as indicated. Highest concentration data point in FP assay excluded from fit due to observed protein precipitation. **C)** Fit of fEMSA data obtained by plotting free dsRNA from gel assay. Data shown are representative of three independent experiments while values are presented as averages with standard deviations.

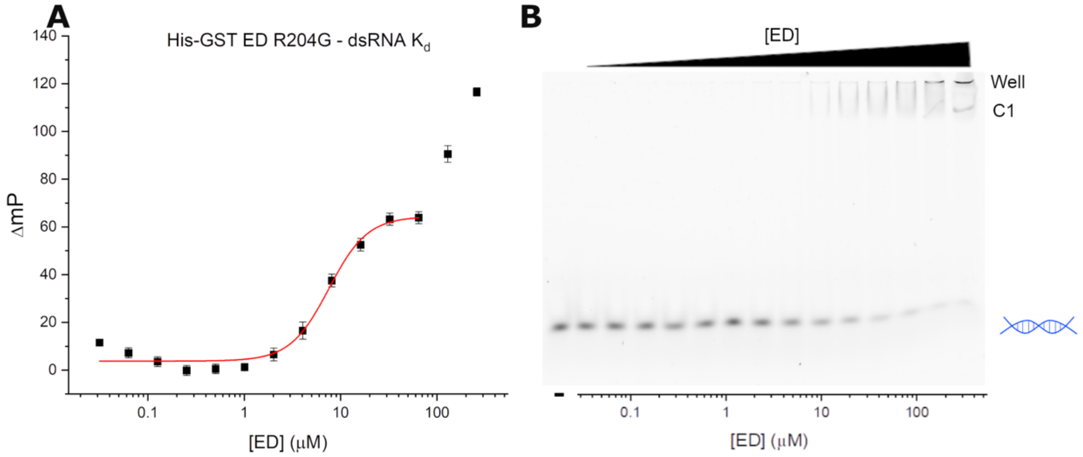

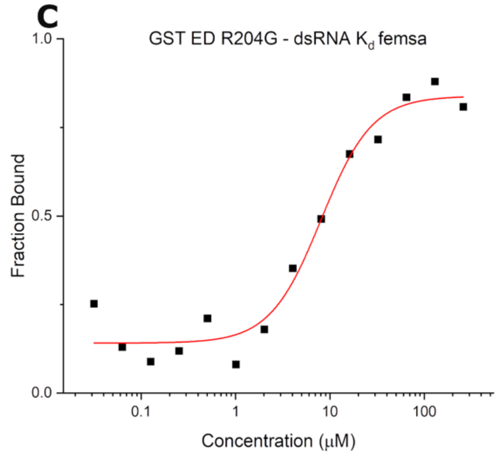

	FP	fEMSA
Average affinity (nM)	8,200 ± 1,000	8,600 ± 1,800
Average R^2	0.97720	0.8695

Figure S19. Binding affinity of Brevig Mission His-GST-ED R204G for dsRNA. A&B) Paired fluorescence polarization data for binding affinity of His-GST-ED R204G (K_d = 8,200 ± 1,000 nM) and fEMSA assay showing increased dsRNA binding with increasing His-GST-ED R204G concentration (K_d = 8,600 ± 1,800 nM). Leftmost lane is RNA only. Free dsRNA is depicted by blue helix on the right, with ED-dsRNA complexes shifted above as indicated. Highest concentration data point in FP assay excluded from fit; binding may be biphasic but is limited by concentration. **C)** Fit of fEMSA data obtained by plotting free dsRNA from gel assay. Data shown are representative of three independent experiments while values are presented as averages with standard deviations.

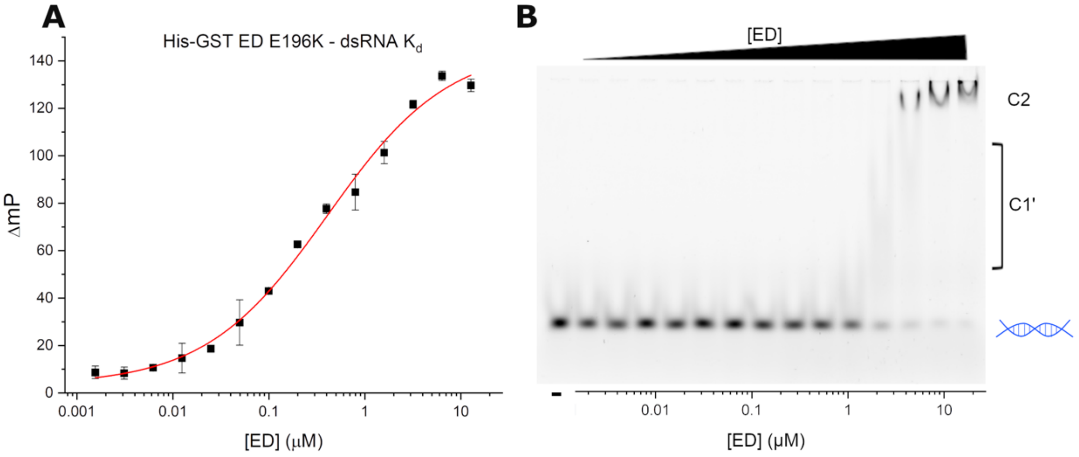

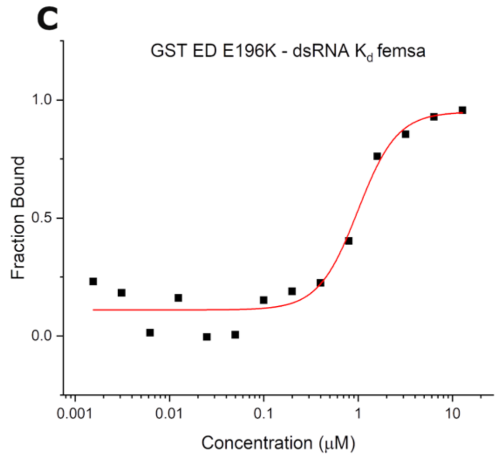

	FP	fEMSA
Average affinity (nM)	480 ± 81	960 ± 180
Average R^2	0.99181	0.94703

Figure S20. Binding affinity of Brevig Mission His-GST-ED E196K for dsRNA. A&B) Paired fluorescence polarization data for binding affinity of His-GST-ED E196K (K_d = 480 ± 81 nM) and fEMSA assay showing increased dsRNA binding with increasing His-GST-ED E196K concentration (K_d = 960 ± 180 nM). Leftmost lane is RNA only. Free dsRNA is depicted by blue helix on the right, with ED-dsRNA complexes shifted above as indicated. **C)** Fit of fEMSA data obtained by plotting free dsRNA from gel assay. Data shown are representative of three independent experiments while values are presented as averages with standard deviations.

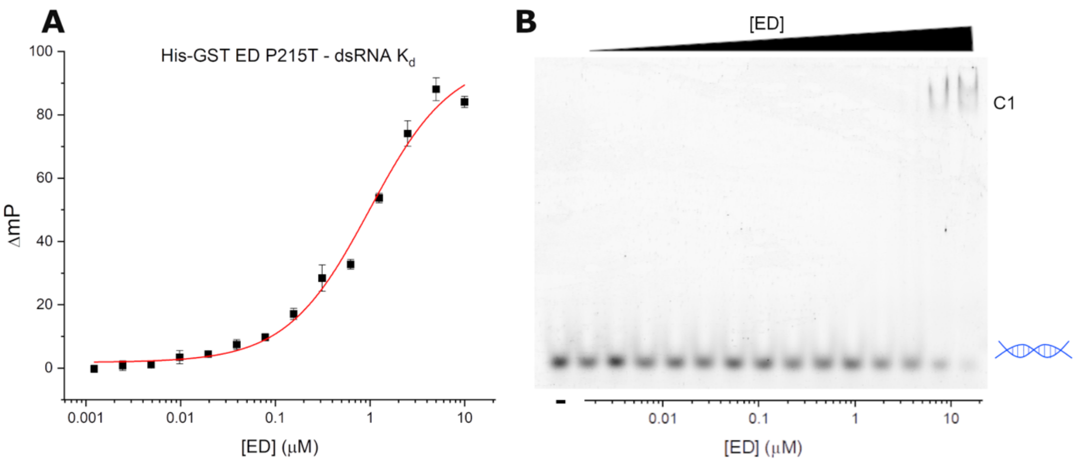

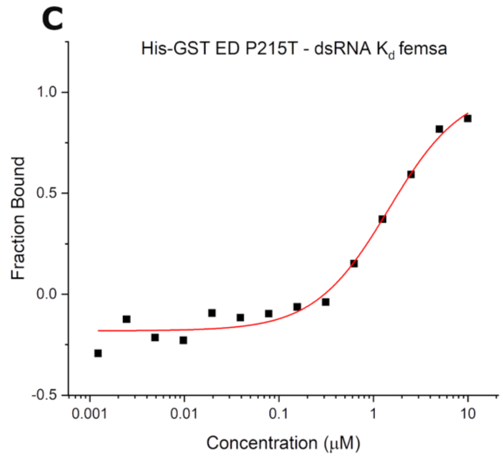

	FP	fEMSA
Average affinity (nM)	1,140 ± 220	1,940 ± 970
Average R^2	0.9836	0.93770

Figure S21. Binding affinity of Brevig Mission His-GST-ED P215T for dsRNA. A&B) Paired fluorescence polarization data for binding affinity of His-GST-ED P215T (K_d = 1,140 ± 220 nM) and fEMSA assay showing increased dsRNA binding with increasing His-GST-ED P215T concentration (K_d = 1,940 ± 970 nM). Leftmost lane is RNA only. Free dsRNA is depicted by blue helix on the right, with ED-dsRNA complexes shifted above as indicated. **C)** Fit of fEMSA data obtained by plotting free dsRNA from gel assay. Data shown are representative of three independent experiments while values are presented as averages with standard deviations.

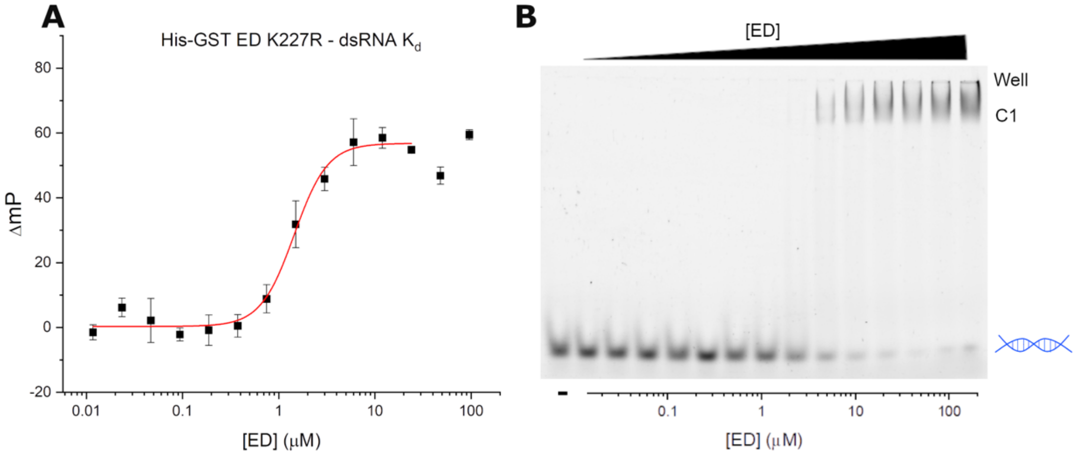

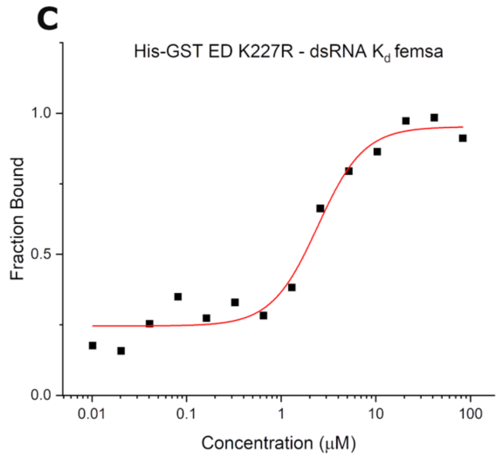

	FP	fEMSA
Average affinity (nM)	1,450 ± 120	1,950 ± 270
Average R^2	0.98773	0.97474

Figure S22. Binding affinity of Brevig Mission His-GST-ED K227R for dsRNA. A&B) Paired fluorescence polarization data for binding affinity of His-GST-ED K227R (K_d = 1,450 ± 120 nM) and fEMSA assay showing increased dsRNA binding with increasing His-GST-ED K227R concentration (K_d = 1,950 ± 270 nM). Leftmost lane is RNA only. Free dsRNA is depicted by blue helix on the right, with ED-dsRNA complexes shifted above as indicated. Highest concentration data point in FP assay excluded from fit due to observed protein precipitation. **C)** Fit of fEMSA data obtained by plotting free dsRNA from gel assay. Data shown are representative of three independent experiments while values are presented as averages with standard deviations.

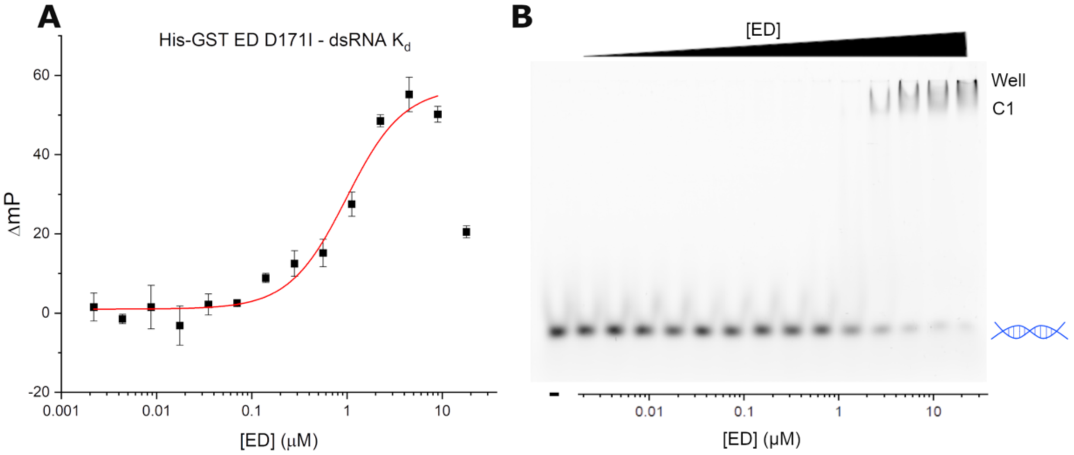

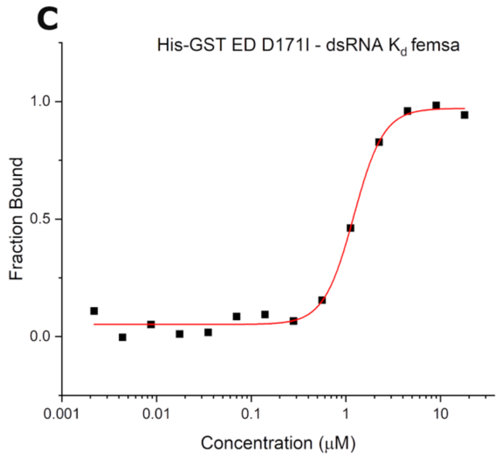

	FP	fEMSA
Average affinity (nM)	950 ± 210	1,140 ± 200
Average R^2	0.97485	0.97720

Figure S23. Binding affinity of Brevig Mission His-GST-ED D171I for dsRNA. A&B) Paired fluorescence polarization data for binding affinity of His-GST-ED D171I (K_d = 950 ± 210 nM) and fEMSA assay showing increased dsRNA binding with increasing His-GST-ED D171I concentration (K_d = 1,140 ± 200 nM). Leftmost lane is RNA only. Free dsRNA is depicted by blue helix on the right, with ED-dsRNA complexes shifted above as indicated. Highest concentration data point in FP assay excluded from fit due to fluorescence quenching. **C)** Fit of fEMSA data obtained by plotting free dsRNA from gel assay. Data shown are representative of three independent experiments while values are presented as averages with standard deviations.

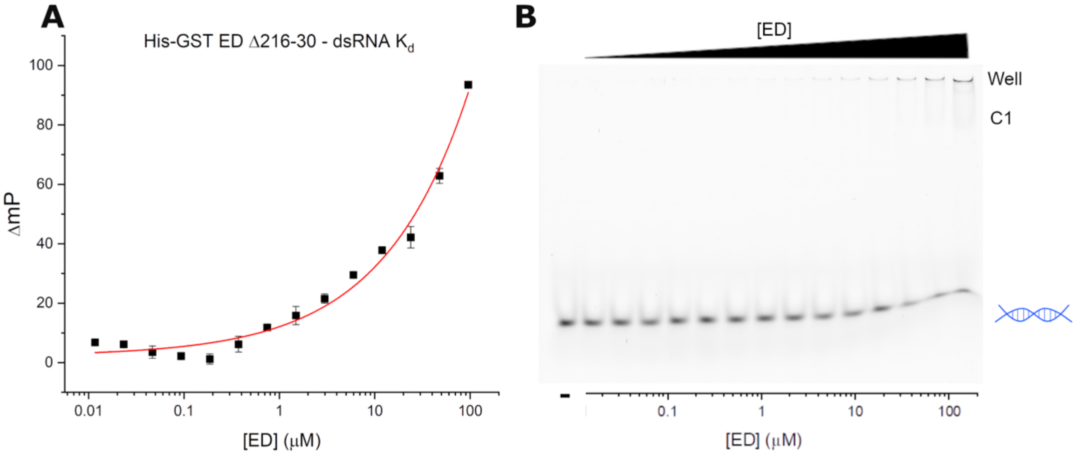

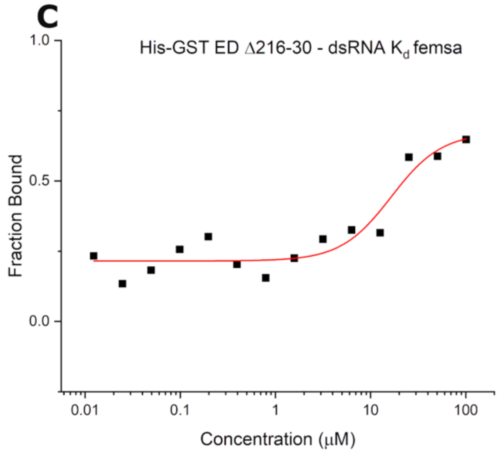

	FP	fEMSA
Average affinity (nM)	>100,000	>100,000
Average R^2	N/A	N/A

Figure S24. Binding affinity of Brevig Mission His-GST-ED Δ216-230 for dsRNA. A&B) Paired fluorescence polarization data for binding affinity of His-GST-ED Δ216-230 (K_d >100,000 nM) and fEMSA assay showing increased dsRNA binding with increasing His-GST-ED Δ216-230 concentration (K_d >100,000 nM). Leftmost lane is RNA only. Free dsRNA is depicted by blue helix on the right, with ED-dsRNA complexes shifted above as indicated. **C)** Fit of fEMSA data obtained by plotting free dsRNA from gel assay. Data shown are representative of three independent experiments while values are presented as averages with standard deviations. Affinities were not able to be accurately assessed due to lack of saturation.

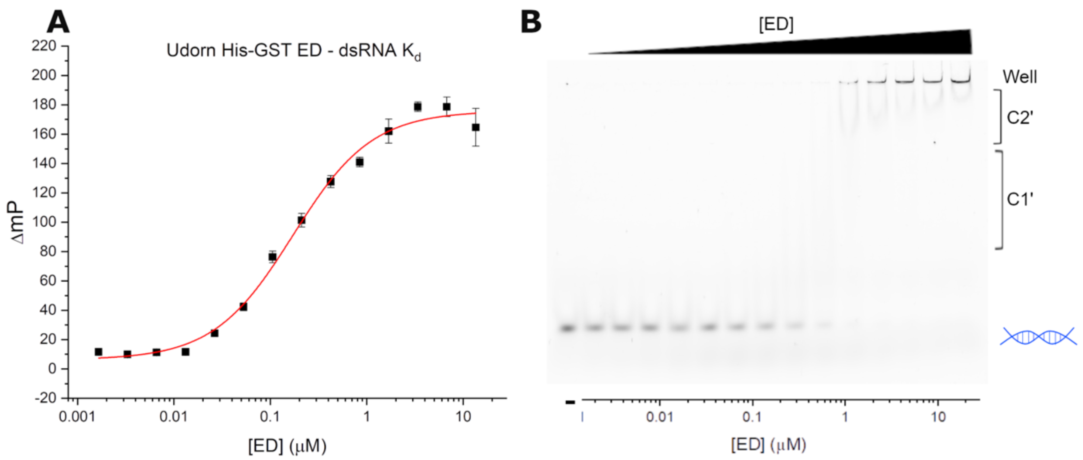

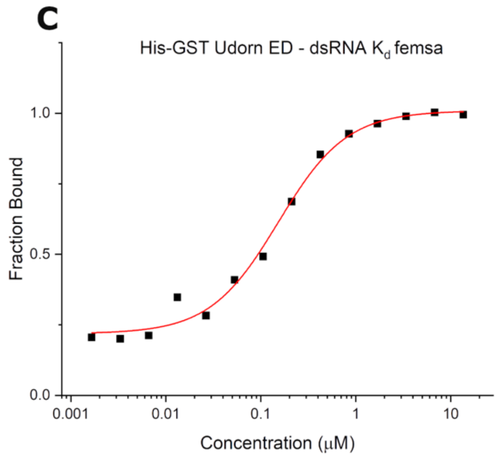

	FP	fEMSA
Average affinity (nM)	180 ± 21	170 ± 35
Average R^2	0.99178	0.97197

Figure S25. Binding affinity of Udorn His-GST-ED for dsRNA. A&B) Paired fluorescence polarization data for binding affinity of Udorn His-GST-ED (K_d = 180 ± 21 nM) and fEMSA assay showing increased dsRNA binding with increasing Udorn His-GST-ED concentration (K_d = 170 ± 35 nM). Leftmost lane is RNA only. Free dsRNA is depicted by blue helix on the right, with ED-dsRNA complexes shifted above as indicated. **C)** Fit of fEMSA data obtained by plotting free dsRNA from gel assay. Data shown are representative of three independent experiments while values are presented as averages with standard deviations.

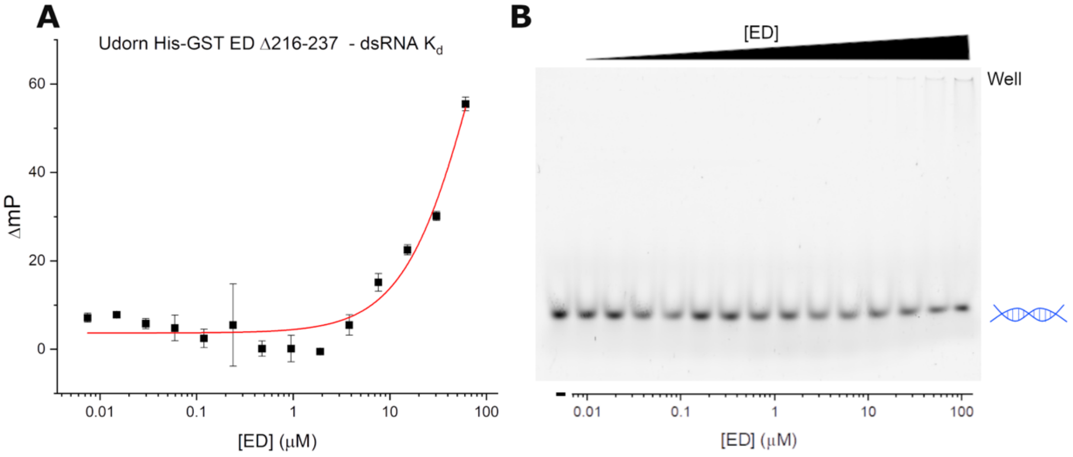

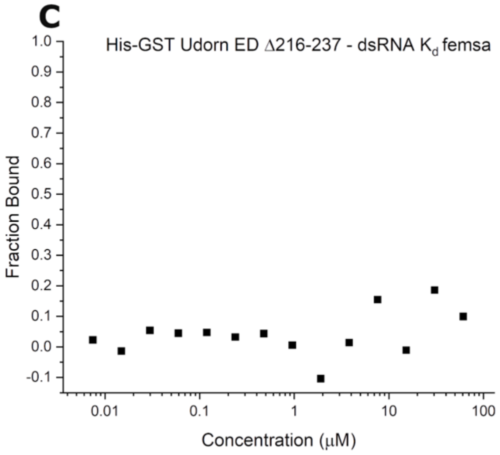

	**FP**	**fEMSA**
Average affinity (nM)	>100,000	>100,000
Average R^2	N/A	N/A

Figure S26. Binding affinity of Udorn His-GST-ED Δ216-237 for dsRNA. A&B) Paired fluorescence polarization data for binding affinity of Udorn His-GST-ED Δ216-237 (K_d >100,000 nM) and fEMSA assay showing increased dsRNA binding with increasing Udorn His-GST-ED Δ216-237 concentration (K_d >100,000 nM). Leftmost lane is RNA only. Free dsRNA is depicted by blue helix on the right, with ED-dsRNA complexes shifted above as indicated. **C)** Fit of fEMSA data obtained by plotting free dsRNA from gel assay. Data shown are representative of three independent experiments. Affinities were not able to be accurately assessed due to lack of saturation.

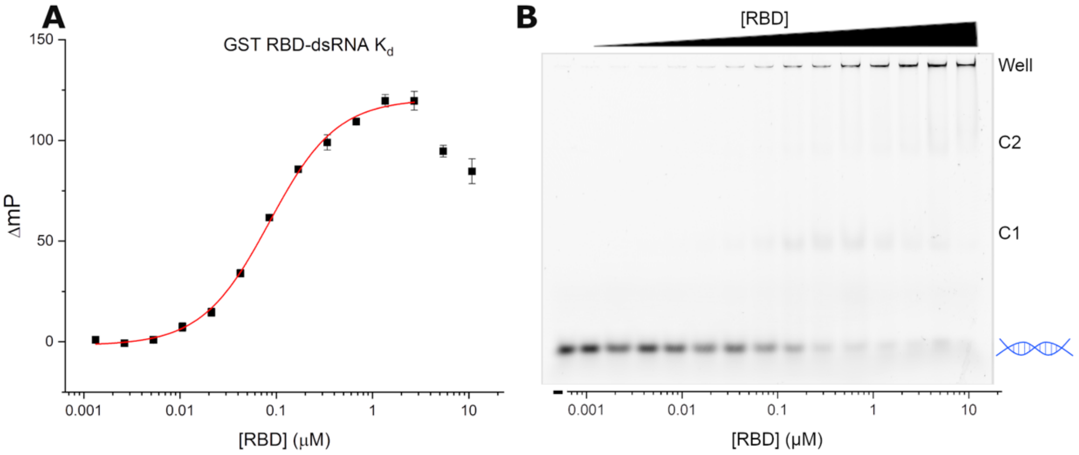

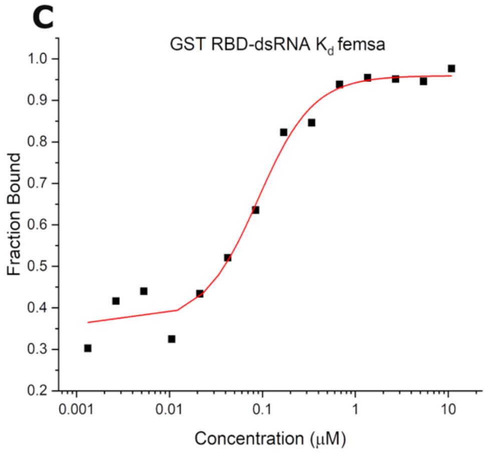

	FP	fEMSA
Average affinity (nM)	76 ± 5	100 ± 32
Average R^2	0.99763	0.94514

Figure S27. Binding affinity of GST-RBD for dsRNA. A&B) Paired fluorescence polarization data for binding affinity of GST-RBD (K_d >100,000 nM) and fEMSA assay showing increased dsRNA binding with increasing GST-RBD concentration (K_d >100,000 nM). Leftmost lane is RNA only. Free dsRNA is depicted by blue helix on the right, with RBD-dsRNA complexes shifted above as indicated. Highest two concentration data points in FP assay excluded from fit due to fluorescence quenching. **C)** Fit of fEMSA data obtained by plotting free dsRNA from gel assay. Data shown are representative of two independent experiments while values are presented as averages with standard deviations.

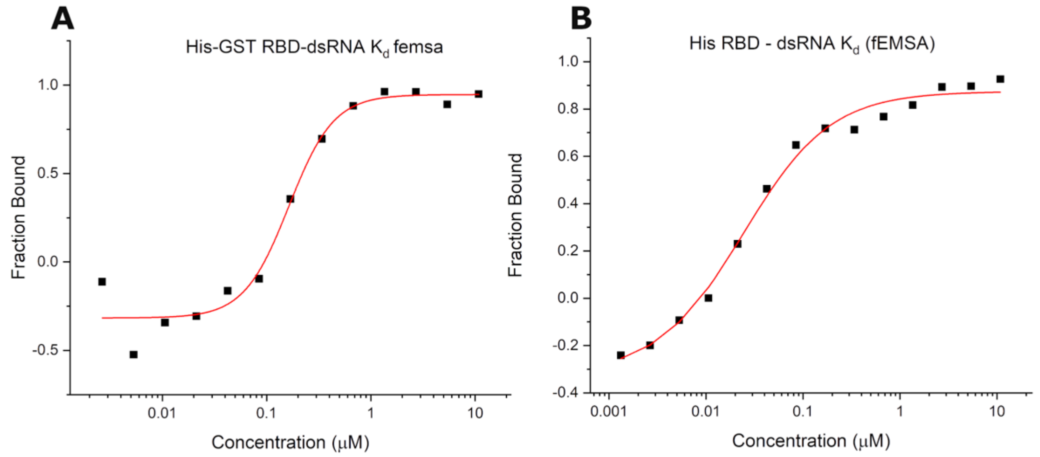

His-GST RBD	FP	fEMSA
Average affinity (nM)	125 ± 9	180 ± 42
Average R^2	0.99724	0.95594

His RBD	FP	fEMSA
Average affinity (nM)	83 ± 11<br>2160 ± 460	55 ± 16
Average R^2	0.9784	0.96969

Figure S28. Binding affinity of Brevig Mission RBD for dsRNA. Fits of fEMSA data obtained by plotting free dsRNA from gel assay for **A)** His-GST-RBD and **B)** His-RBD. Data shown are representative of three independent experiments while values are presented as averages with standard deviations.

Protein Purification Information for Table 1 (Figure S29)

His-GST RBD

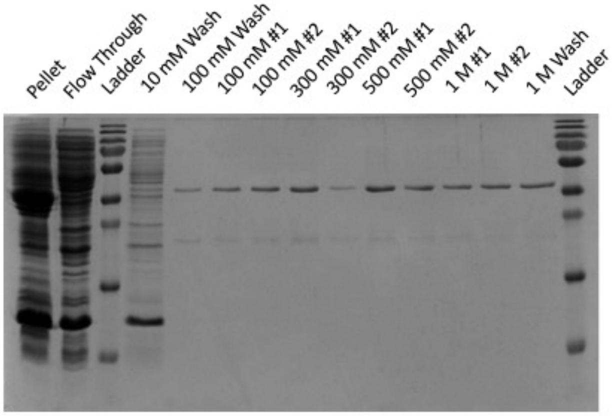

260/280: 0.75

His-GST RBD R21Q

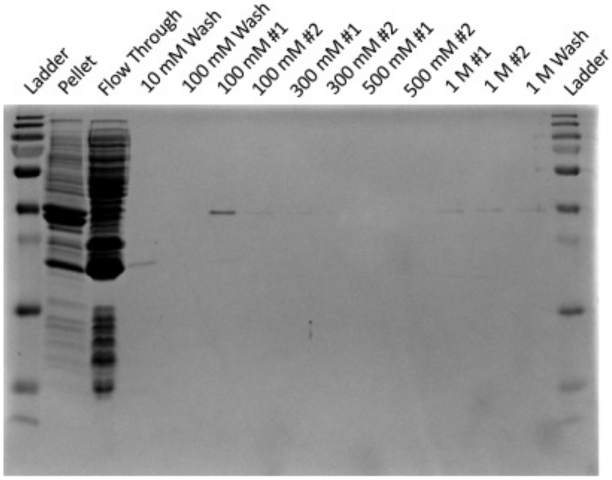

260/280: 0.90

His-GST RBD F22V

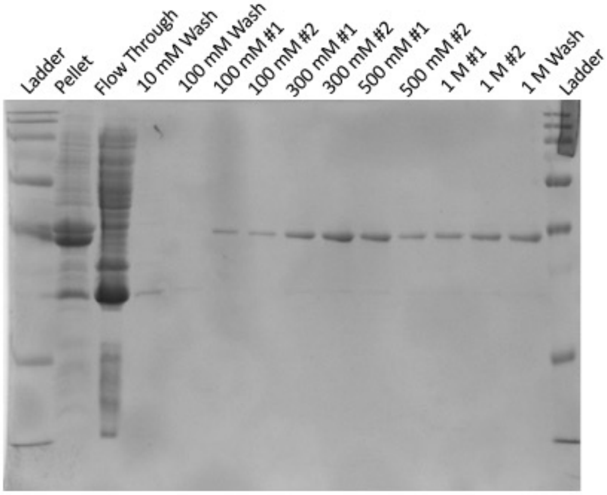

260/280: 0.77

His-GST RBD R38A/K41A

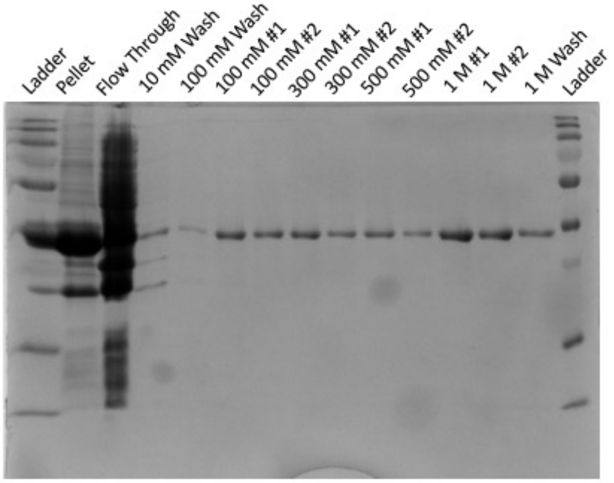

260/280: 0.66

His-GST RBD D53N

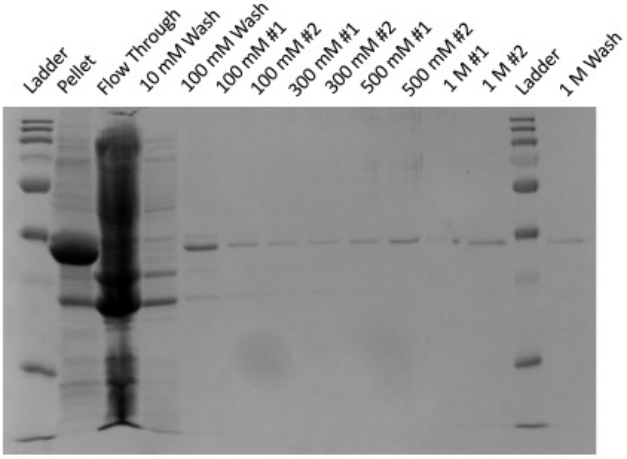

260/280: 1.32

His-GST RBD R59H

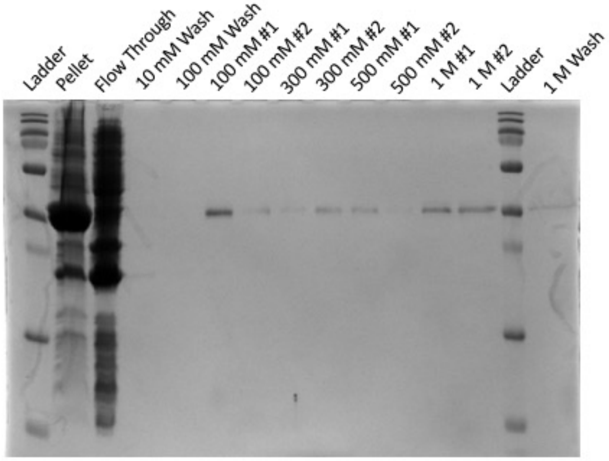

260/280: 0.87

His-GST RBD T49V

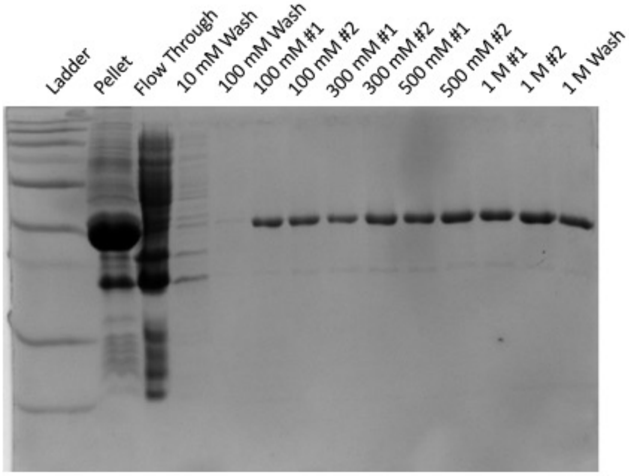

260/280: 0.80

His-GST RBD T56A

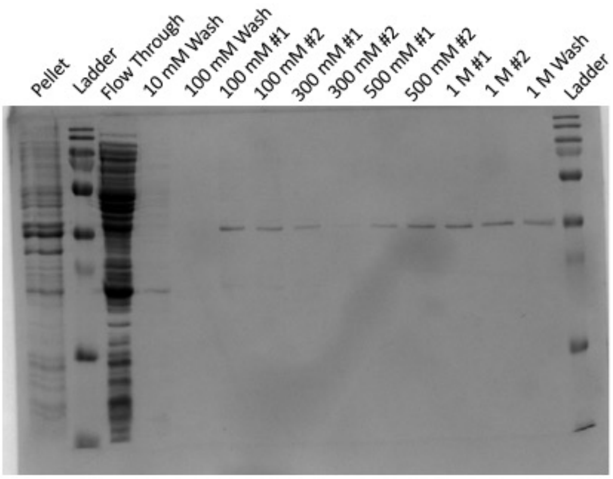

260/280: 1.43

His-GST RBD R35A

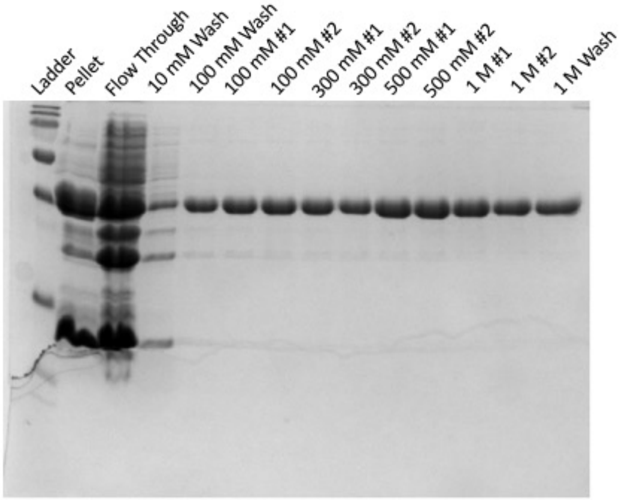

260/280: 0.74

His-GST NS1A R35A

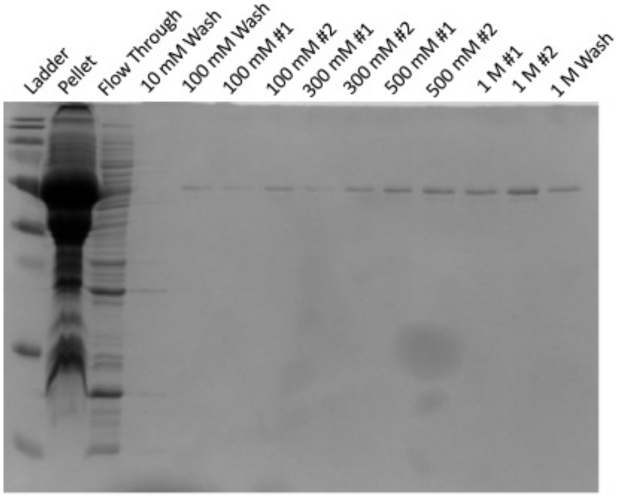

260/280: 0.86

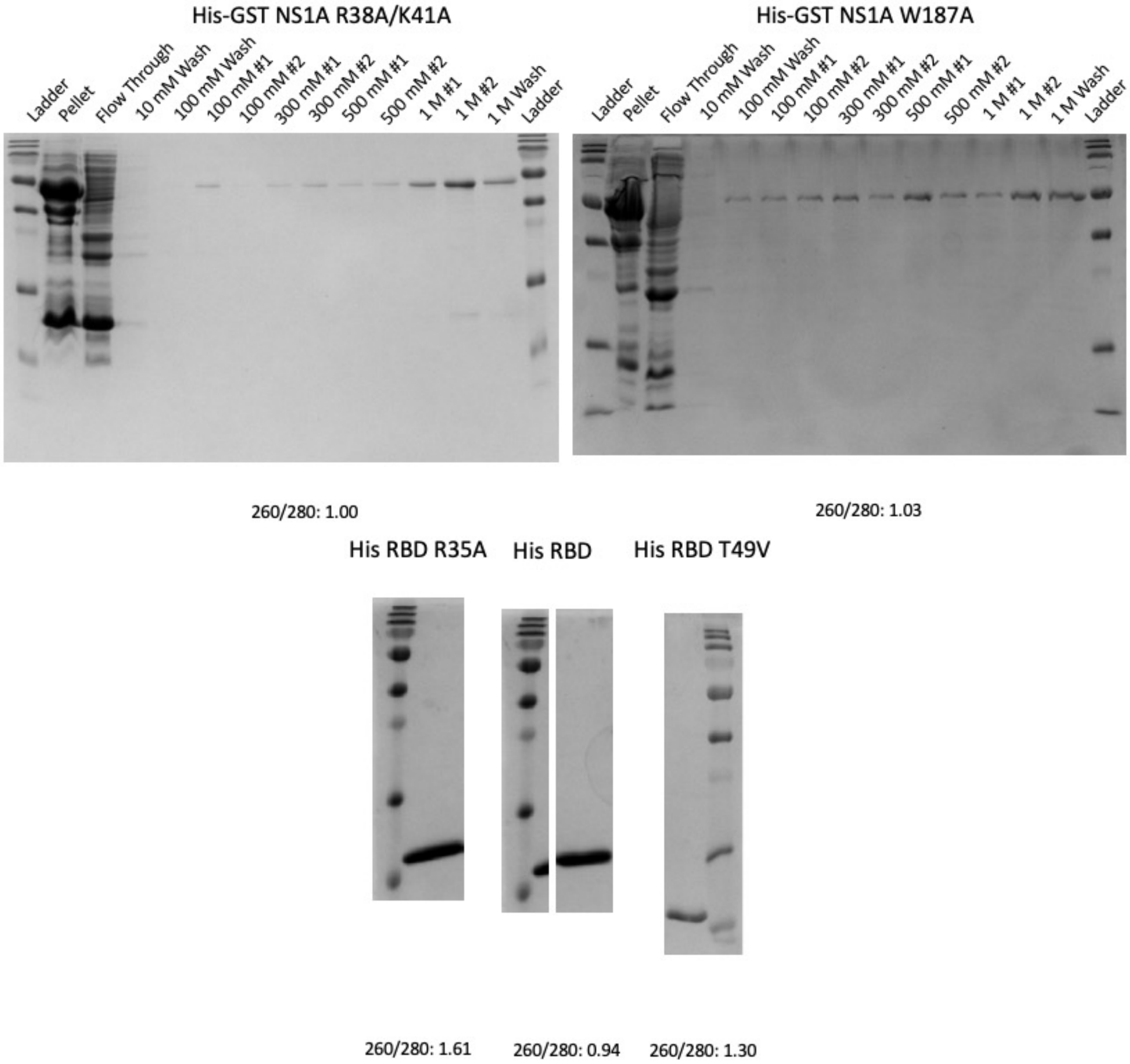

Figure S29. SDS-PAGE gels assessing protein purity for all proteins used in FP and fEMSA assays shown in Table 1. Each fraction from the purification process was run on a 15% or 18% SDS-PAGE gel and stained with Coomassie, and purity was assessed using Bio-Rad Image Lab. Only fractions determined to be of 95% purity or greater were pooled and buffer exchanged into storage buffer for use in assays. His-tag only constructs required further purification via size exclusion chromatography and were reassessed after fraction pooling.

Protein Purification Information for Table 2 (Figure S30)

His-GST ED

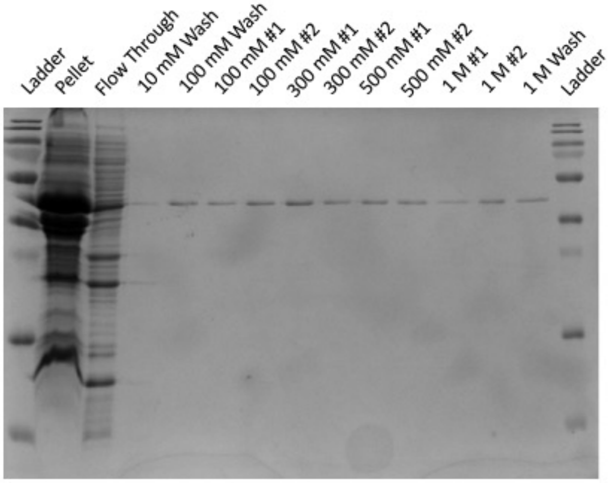

260/280: 0.69

His-GST ED M98L

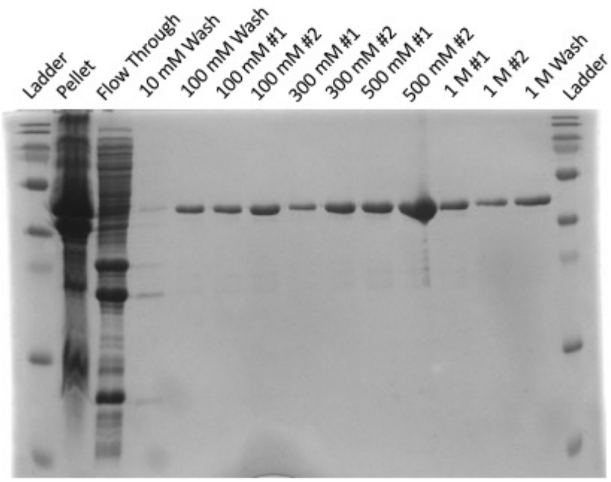

260/280: 0.65

His-GST ED S114P

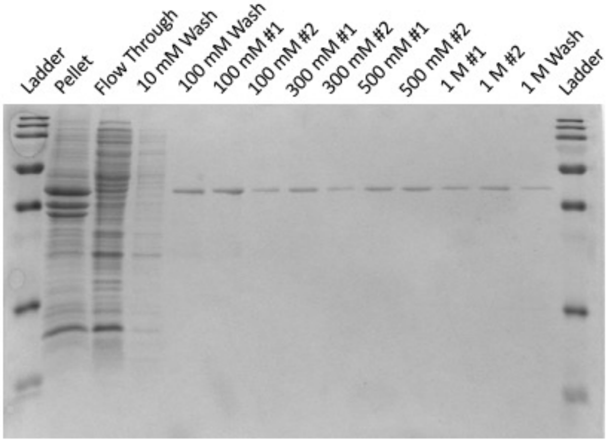

His-GST ED M119I

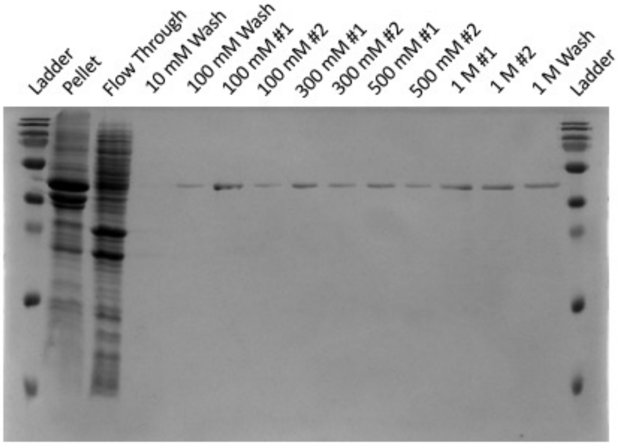

260/280: 0.84

260/280: 0.71

His-GST ED D171I

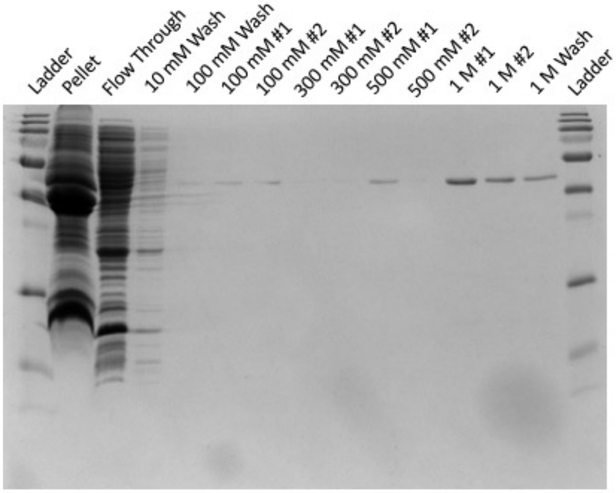

260/280: 0.69

His-GST ED W187A

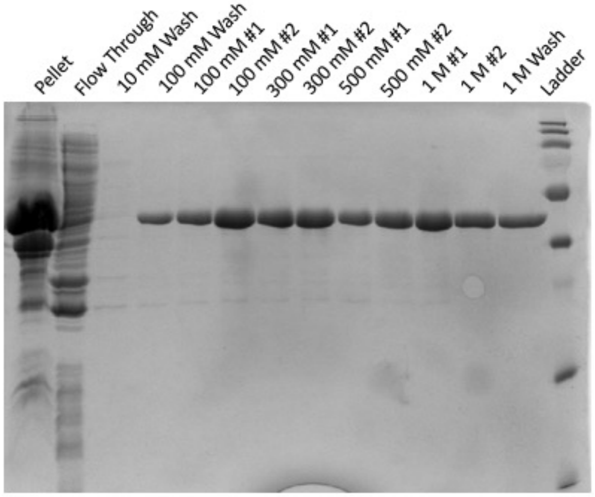

260/280: 0.60

His-GST ED E196K

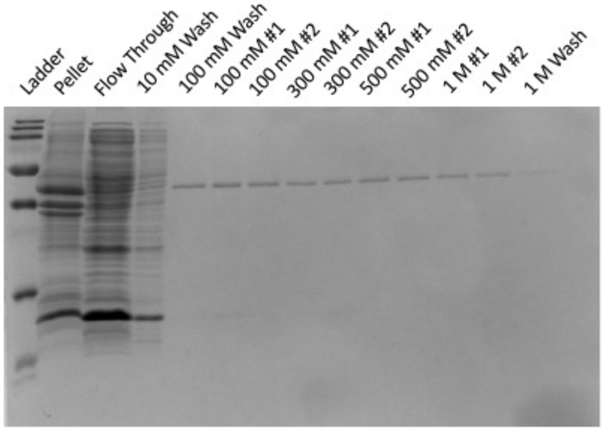

260/280: 0.68

His-GST ED R204G

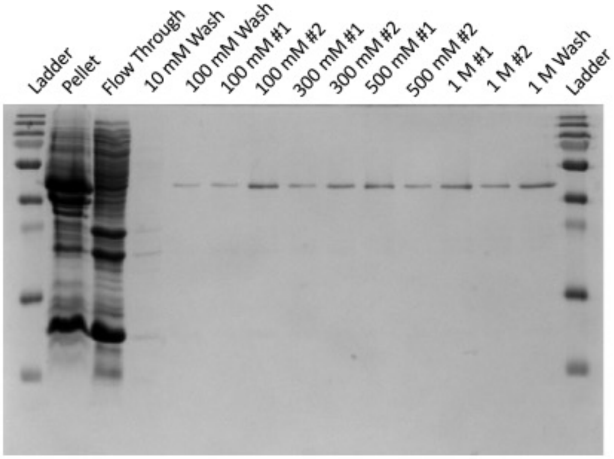

260/280: 0.62

His-GST ED P215T

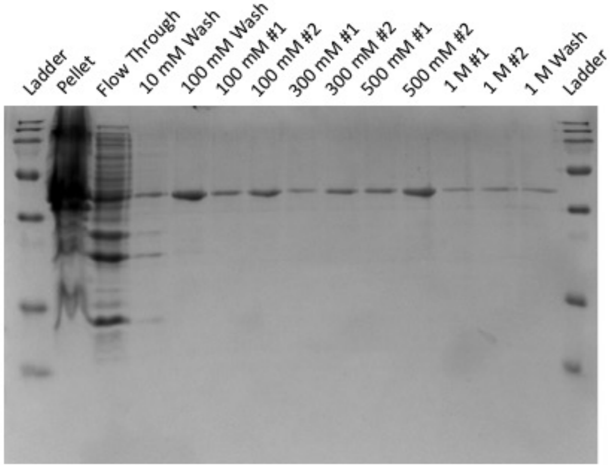

260/280: 0.71

His-GST ED K227R

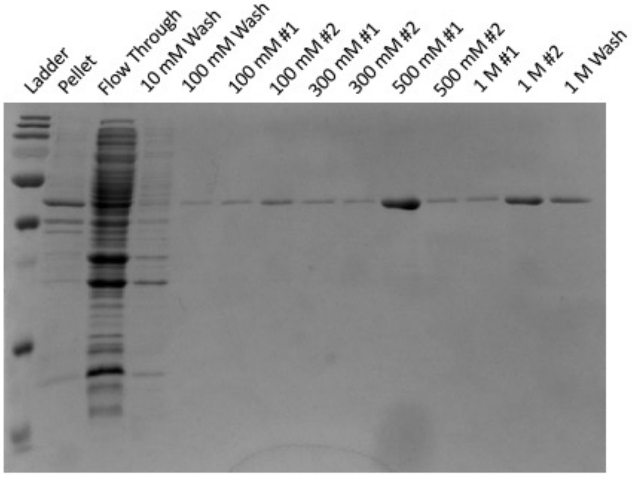

260/280: 0.73

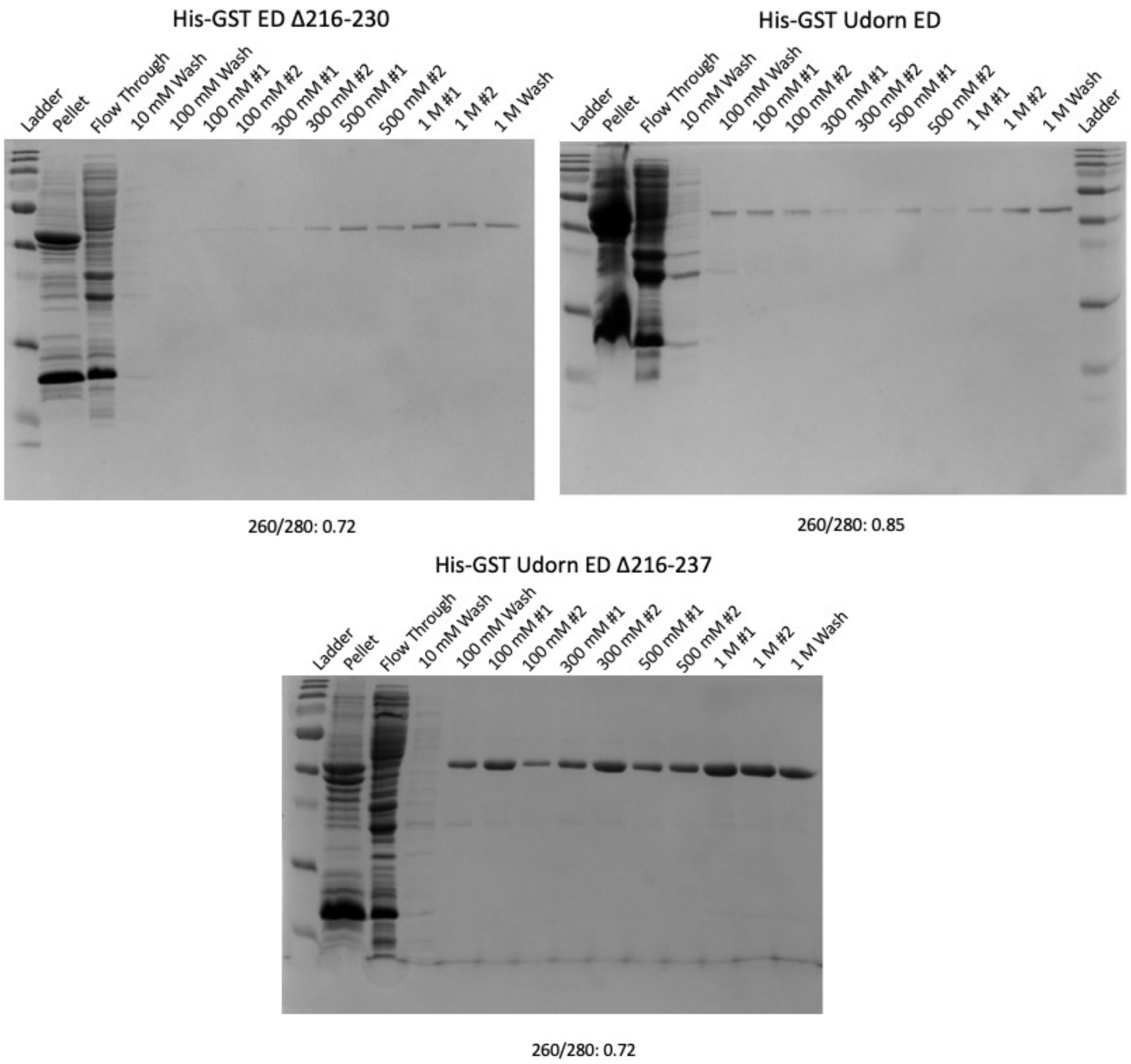

Figure S30. SDS-PAGE gels assessing protein purity for all proteins used in FP and fEMSA assays shown in Table 2. Each fraction from the purification process was run on a 15% or 18% SDS-PAGE gel and stained with Coomassie, and purity was assessed using Bio-Rad Image Lab. Only fractions determined to be of 95% purity or greater were pooled and buffer exchanged into storage buffer for use in assays. His-tag only constructs required further purification via size exclusion chromatography and were reassessed after fraction pooling.

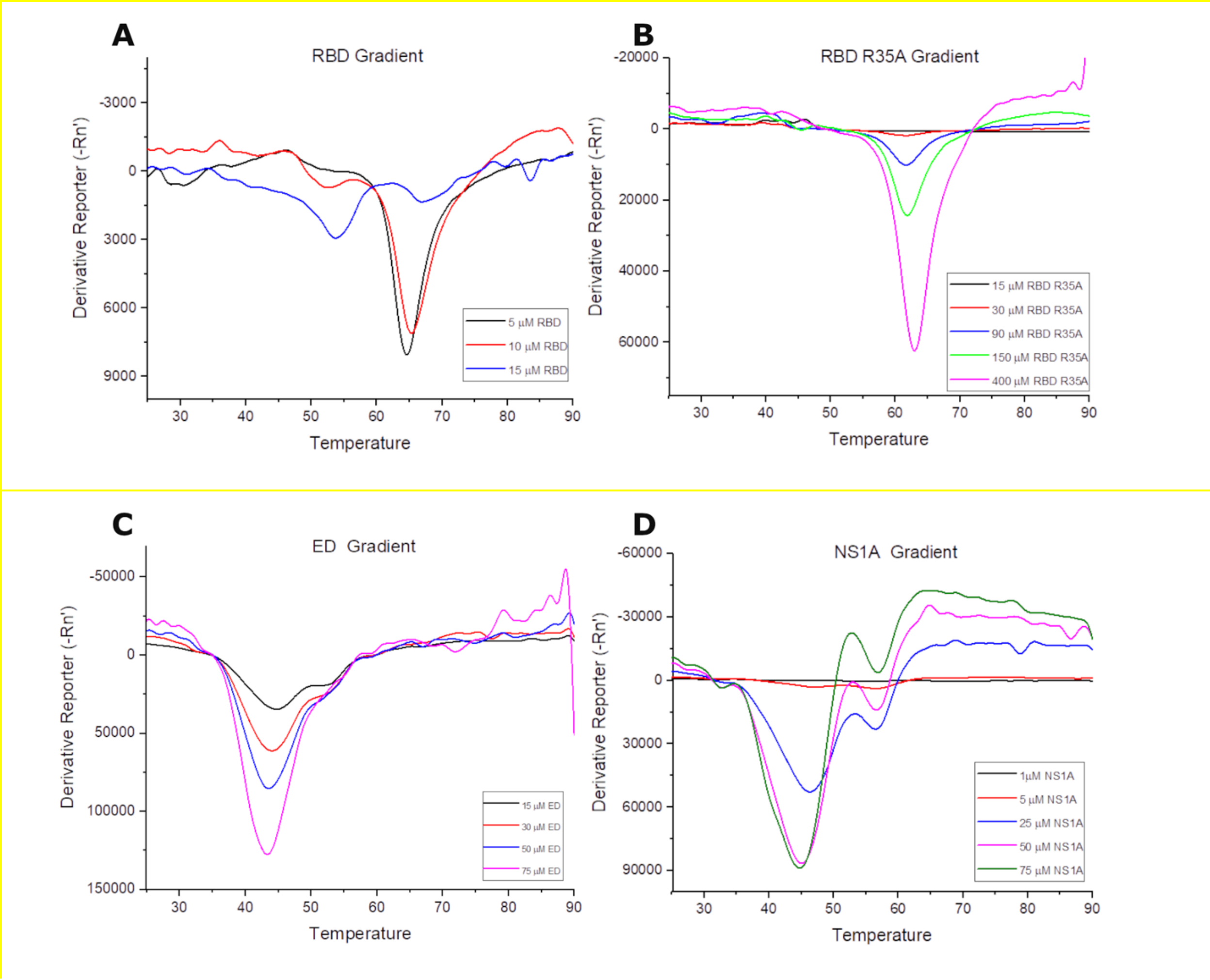

Figure S31. Thermal Shift Assay (TSA) of increasing concentration of His-tagged NS1A Constructs. A) His-RBD TSA with a Tm1 of 53.4 ± 0.7 °C, and a Tm2 around 63 °C indicating dimer formation as concentration increases. **B)** His-RBD R35A TSA with a single average Tm of 61.4 ± 0.2 °C at all concentrations up to 400 μM, indicating that it is dimer defunct. As concentration increased, this value varied from 59.3 °C to 63.2 °C. **C)** His-ED TSA with a Tm1 of around 43 °C and a Tm2 of 51.7 ± 0.1 °C, the former of which varied slightly from 42.9-44.6 °C, increasing as concentration (and likely therefore dimer stability) increased. **D)** His-NS1A R38A/K41A TSA with a Tm1 which appeared to correlate to disruption of the ED dimer (44.8 – 46.8 °C) and Tm2 at 56.5 °C, which may be due to both RBD dimer disruption as well as protein unfolding. While not indicative of homodimer affinity, these results indicate that the WT RBD and ED are capable of dimer formation, while the RBD R35A mutant construct is not capable of dimer formation.

Impact of Varying A260/A280 Values on Binding Affinities (Figure S32)

A

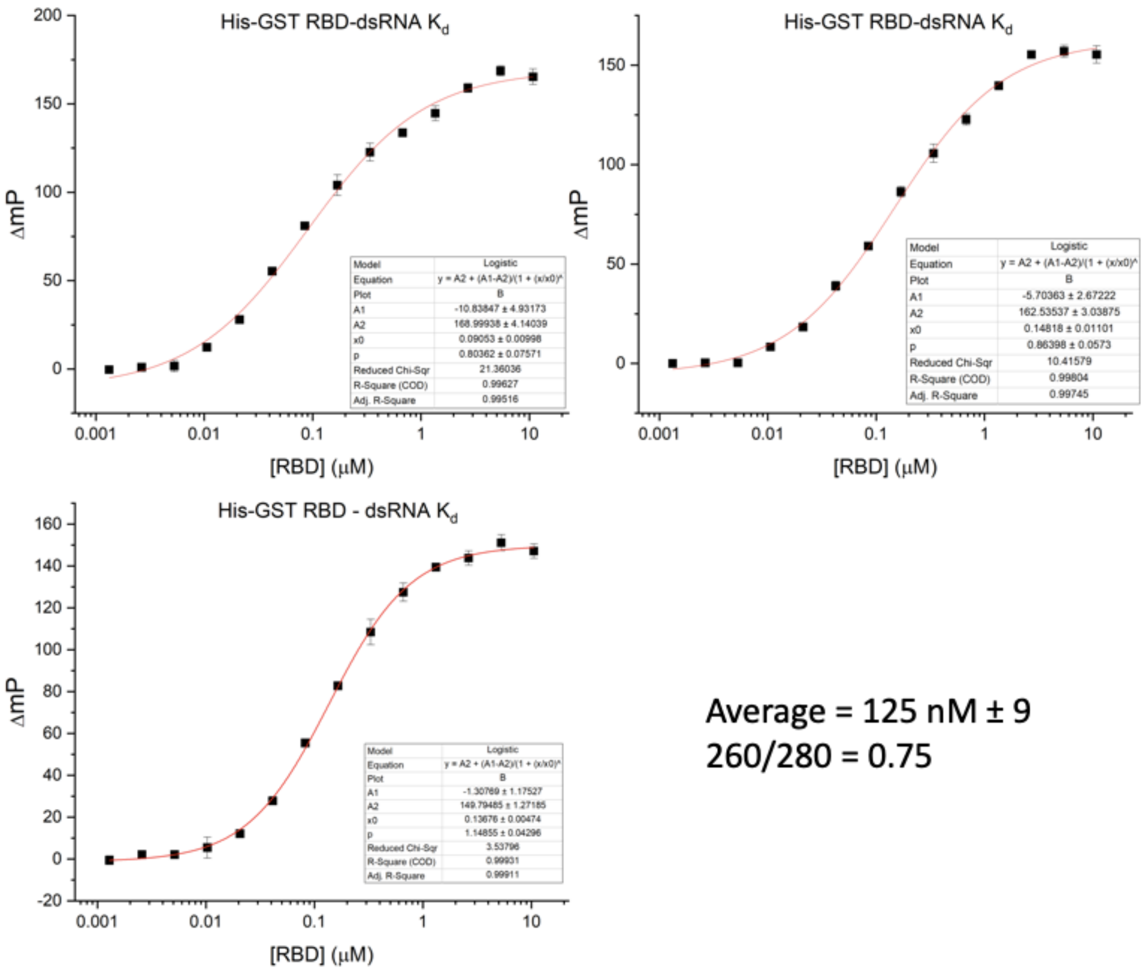

Average = 125 nM ± 9
260/280 = 0.75

B

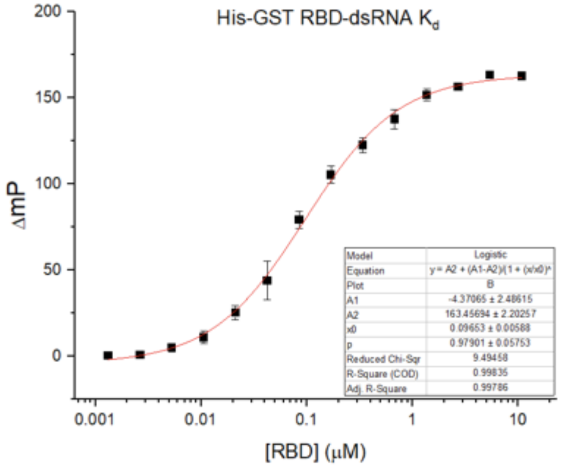

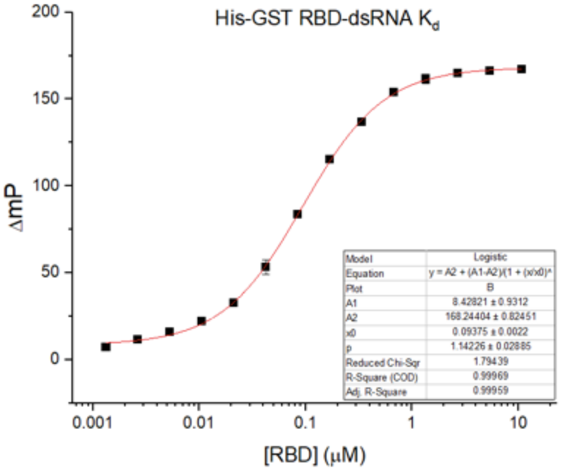

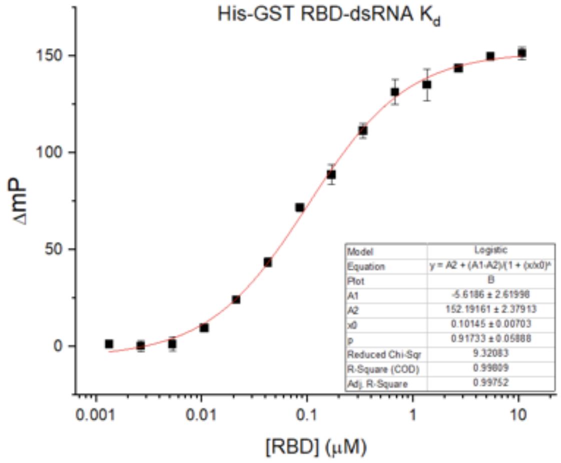

Average = 97 nM ± 5.4
260/280 = 0.99

C

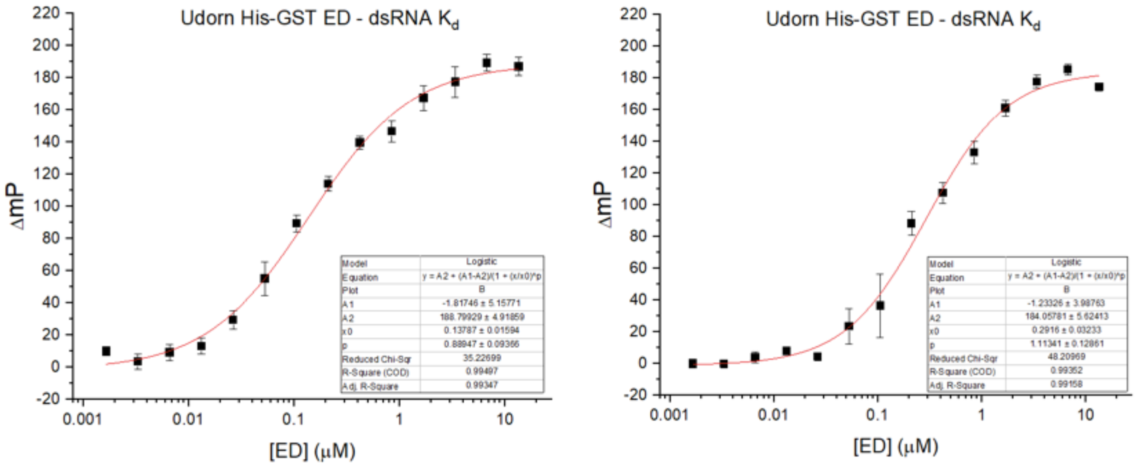

Average = 215 nM ± 25
260/280 = 0.76

D

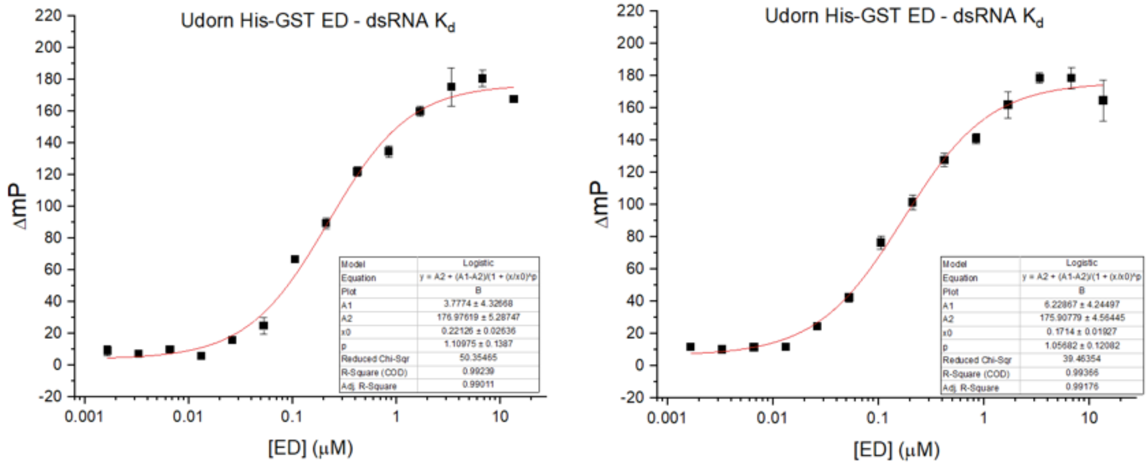

Average = 196 nM ± 23
260/280 = 0.85

E

Construct	A260/A280	Binding Affinity
His-GST RBD – Prep A	0.75	125 nM ± 9
His-GST RBD – Prep B	0.99	97 nM ± 5
His-GST Udorn ED – Prep A	0.76	215 nM ± 25
His-GST Udorn ED – Prep B	0.85	196 nM ± 23

Figure S32. Comparison of Fluorescence Polarization data obtained from separate protein preparations with varying A260/A280 values. A) His-GST-RBD with an A260/280 ratio of 0.75 with an average affinity of 125 nM ± 9. **B)** His-GST-RBD with an A260/280 ratio of 0.99 with an average affinity of 97 nM ± 5. **C)** His-GST-Udorn ED with an A260/280 ratio of 0.76 with an average affinity of 215 nM ± 25. **D)** His-GST-Udorn ED with an A260/280 ratio of 0.85 with an average affinity of 196 nM ± 23. **E)** Summarized data for compared protein preparations provided at the bottom.

Supplemental Information Experimental Methods

Thermal Shift Assays (Figure S31)

Protein was diluted in thermal stability buffer (25 mM HEPES pH 7.0, 150 mM NaCl) to the final desired concentrations and 16 µL was added to the Thermo AB-1384 (PCR Plate, 384-well, standard). SYPRO™ Orange (5,000 x stock) was diluted 1:200 (1:100 for RBD constructs) and 4 µL was added to each well, and was sealed with Thermo AB-1170 (optical clear adhesive seal). The plate was spun down for 2 min at 1,000 rpm and was heated from 20 to 95 °C. Data were processed using Origin Pro 2021b where the derivative of the signal was calculated and smoothed using 35 points of FFT filter. Tm values were calculated using the peak analyzer function. All ED and RBD assays were replicated 3 times. Full-length assays were replicated 3 times up to 5 µM, but only once for high concentrations.

Table S1. Primers used for mutagenesis. "*" Indicates overlap with R38A, used after R38A primer for double mutant. Same primers were used for mutagenesis of the full-length protein.

**RBD R21Q**	Forward	TGTGCGTAAACAGTTTGCGGACCAAGAAC
	Reverse	TGCCACAGGAAGCAGTCC
**RBD F22V**	Forward	GCGTAAACGCGTGGCGGACCAAG
	Reverse	ACATGCCACAGGAAGCAG
**RBD R35A**	Forward	GTTTCTGGATGCTCTGCGTCGTGATCAGAAGAG
	Reverse	GGCGCATCACCCAGTTCT
**RBD R38A**	Forward	TCGTCTGCGTGCCGATCAGAAGAGCC
	Reverse	TCCAGAAACGGCGCATCA
**RBD K41A***	Forward	TGCCGATCAGGCCAGCCTGCGTG
	Reverse	CGCAGACGATCCAGAAAC
**RBD T49V**	Forward	TCGCGGTTCTGTGCTGGGCCTGG
	Reverse	CCACGCAGGCTCTTCTGA
**RBD D53N**	Forward	CCTGGGCCTGAATATTGAAACCG
	Reverse	GTAGAACCGCGACCACGC
**RBD T56A**	Forward	GGATATTGAAGCGGCGACGCGTGC
	Reverse	AGGCCCAGGGTAGAACCG
**RBD R59H**	Forward	AACCGCGACGCATGCCGGTAAAC
	Reverse	TCAATATCCAGGCCCAGGGTAG
**ED M98L**	Forward	GCTGGAAGAACTGAGTCGTGACT
	Reverse	GTCATATCGGTCAGATAGC
**ED S114P**	Forward	GGTGGCAGGCCCGCTGTGCATTC
	Reverse	TTCTGTTTCGGCATCAGCATAAAC
**ED M119I**	Forward	GTGCATTCGCATTGATCAAGCTATCATGG
	Reverse	AGGGAGCCTGCCACCTTC
**ED D171I**	Forward	GGGTCATACCATTGAAGACGTCAAAAACGCCGTGGG TGTTC
	Reverse	GGCAGCGACGGCAGCGGT
**ED W187A**	Forward	CGGTCTGGAAGCGAACGATAATACCG
	Reverse	CCAATCAGAACACCCACG
**ED E196K**	Forward	CCGTGTGTCTAAAACGCTGCAGC
	Reverse	ACGGTATTATCGTTCCATTCCAG
**ED R204G**	Forward	TTTCGCATGGGGCAGCTCTAACG
	Reverse	CGCTGCAGCGTTTCAGAC
**ED P215T**	Forward	CCCGCCGCTGACCCCGAAGCAAA
	Reverse	CGACCATTTTCGTTAGAGCTGCG
**ED K227R**	Forward	TCGCACGATCCGTTCTGAAGTGTAAG
	Reverse	GCCATTTTGCGTTTTTGC
**ED Δ216-230**	Forward	TAAGAGCTCCCGCTGAGCAATAACTAGCATAACCCC
	Reverse	CGGCAGCGGCGGGCGACC
**Udorn ED Δ216-237**	Forward	TAAAGCAGCGGCCATATCGAAGG
	Reverse	GGTCAAAGGCGGACGACC
